# Combined miRNA and mRNA sequencing reveals the defensive strategies of resistant YHY15 rice against differentially virulent brown planthoppers

**DOI:** 10.3389/fpls.2024.1366515

**Published:** 2024-03-18

**Authors:** Bin Yu, Mengjia Geng, Yu Xue, Qingqing Yu, Bojie Lu, Miao Liu, Yuhan Shao, Chenxi Li, Jingang Xu, Jintao Li, Wei Hu, Hengmin Tang, Peng Li, Qingsong Liu, Shengli Jing

**Affiliations:** ^1^ College of Life Sciences, Xinyang Normal University, Xinyang, China; ^2^ Hubei Provincial Key Laboratory for Protection and Application of Special Plant Germplasm in Wuling Area of China, College of Life Sciences, South-Central Minzu University, Wuhan, China; ^3^ Guangdong Provincial Key Laboratory of New Technology in Rice Breeding, Rice Research Institute, Guangdong Academy of Agricultural Sciences, Guangzhou, China; ^4^ State Key Laboratory of Cotton Bio-breeding and Integrated Utilization, State Key Laboratory of Crop Stress Adaptation and Improvement, Key Laboratory of Plant Stress Biology, School of Life Sciences, Henan University, Kaifeng, China

**Keywords:** rice, *Bph15*, brown planthopper, virulent populations, resistance mechanism

## Abstract

**Introduction:**

The brown planthopper (BPH) poses a significant threat to rice production in Asia. The use of resistant rice varieties has been effective in managing this pest. However, the adaptability of BPH to resistant rice varieties has led to the emergence of virulent populations, such as biotype Y BPH. YHY15 rice, which carries the BPH resistance gene *Bph15*, exhibits notable resistance to biotype 1 BPH but is susceptible to biotype Y BPH. Limited information exists regarding how resistant rice plants defend against BPH populations with varying levels of virulence.

**Methods:**

In this study, we integrated miRNA and mRNA expression profiling analyses to study the differential responses of YHY15 rice to both avirulent (biotype 1) and virulent (biotype Y) BPH.

**Results:**

YHY15 rice demonstrated a rapid response to biotype Y BPH infestation, with significant transcriptional changes occurring within 6 hours. The biotype Y-responsive genes were notably enriched in photosynthetic processes. Accordingly, biotype Y BPH infestation induced more intense transcriptional responses, affecting miRNA expression, defenserelated metabolic pathways, phytohormone signaling, and multiple transcription factors. Additionally, callose deposition was enhanced in biotype Y BPH-infested rice seedlings.

**Discussion:**

These findings provide comprehensive insights into the defense mechanisms of resistant rice plants against virulent BPH, and may potentially guide the development of insect-resistant rice varieties.

## 1 Introduction

Rice (*Oryza sativa* L.) was domesticated approximately 10,000 years ago in the lower Yangtze Valley in China. From there, it spreads across Asia, Africa, Europe, and the Americas, and now serves as a staple crop for more than half of the world’s population (Cheng et al., 2013; Gutaker et al., 2020). However, rice production suffers from numerous pests and pathogens. Among them, the brown planthopper (BPH; *Nilaparvata lugens* Stål) is considered extremely destructive (Bottrell and Schoenly, 2012; Cheng et al., 2013; Jing et al., 2017). BPH feeds on phloem sap and causes dwarfing, wilting, browning, drying, and ultimately death in severe cases (Wang et al., 2008). In addition, BPH serves as a vector for viral diseases, resulting in significant yield shortfalls and economic losses (Cheng et al., 2013).

Over the course of an evolutionary arms race between these two species, rice has developed sophisticated defensive mechanisms against BPH (Cheng et al., 2013), including both basic defenses and resistance (R) gene-mediated defenses. To date, approximately 40 major BPH resistance genes have been identified in cultivated and wild rice species (Wang et al., 2023). Molecular cloning and functional characterization of BPH resistance genes have clarified the molecular mechanisms of rice resistance to BPH (Zheng et al., 2021). One mechanism involves the occlusion of sieve tubes with callose, which is a common plant defense against sap-sucking insects. This mechanism is the most effective in rice varieties carrying BPH resistance genes such as B5 (carrying *Bph14* and *Bph15*), RI35 (carrying *Bph14*), and YHY15 (carrying *Bph15*) (Hao et al., 2008). In susceptible varieties such as Taichung Native1 (TN1), β-1,3-glucanases (which are only weakly induced in resistant plants) decompose the deposited callose and thereby facilitate continuous feeding by BPH (Hao et al., 2008). *Bph14* encodes a typical CC-NB-LRR protein which interacts with the transcription factors (TFs) OsWRKY46 and OsWRKY72 to increase the expression of the receptor-like cytoplasmic kinase gene *RLCK281* and the callose synthase gene by binding to their promoters (Du et al., 2009; Hu et al., 2017). *Bph15* is located on the short arm of chromosome 4 and is composed of a gene cluster encoding three plant lectin receptor-like kinase proteins (LecRLKs) (Yang et al., 2004; Lv et al., 2014; Xiao et al., 2016). However, the precise mechanism by which *Bph15* confers resistance to BPH is unknown.

Phytohormones are thought to play pivotal roles in the interaction of rice plants and BPH herbivory, including salicylic acid (SA), ethylene (ET), jasmonic acid (JA), cytokinin (CK), brassinosteroid (BR), and abscisic acid (ABA). The SA pathway contributes to the immune response against piercing–sucking insects and is involved in *Bph6-*, *Bph9-*, *Bph14-*, and *Bph29-*mediated resistance in rice (Du et al., 2009; Wang et al., 2015; Zhao et al., 2016; Hu et al., 2017; Guo et al., 2018). Antagonistically, ET negatively regulates BPH resistance in rice (Lu et al., 2011, 2014; Ma et al., 2020). However, the role of JA in rice resistance to BPH remains controversial as the silencing of different genes related to JA biosynthesis and signaling results in diverse impacts on BPH resistance. For example, silencing *9-lipoxygenase* (*OsLOX9*/*OsHI-LOX*, a JA biosynthesis-related gene) enhances BPH resistance, suggesting that JA negatively regulates BPH resistance (Zhou et al., 2009). On the other hand, silencing *coronatine insensitive 1* (*OsCOI1*, a JA receptor gene) has no effect on BPH resistance (Ye et al., 2012). According to studies in allene oxide cyclase (AOC, a JA biosynthesis-related enzyme)- and Myelocytomatosis protein 2 (MYC2, a bHLH TF in the JA pathway)-knockout mutant rice, JA appears to be a positive regulator of BPH resistance (Xu et al., 2021). The exogenous application of JA similarly suggests this (Guo et al., 2018). Recent research also suggests that CK may positively regulate BPH resistance in a JA-dependent manner (Zhang et al., 2022). Conversely, BR promotes BPH susceptibility by modulating SA and JA signaling (Pan et al., 2018). ABA enhances BPH resistance by promoting callose formation (Dinh et al., 2013; Liu et al., 2017) and synergizes with JA to stimulate the expression of TFs in BPH-infested rice (Li et al., 2022). Furthermore, a coordinated CK, SA, and JA signaling network has been found to be activated in *Bph6*-near isogenic lines (NILs) (Guo et al., 2018). Taken together, these findings suggest that the various phytohormones play diverse roles in the BPH defense response, and that there is complex crosstalk between them.

BPH biotypes with increased virulence have emerged in response to pressures imposed by these defense mechanisms, which are capable of overcoming resistance conferred by major resistance genes. Biotype 1 BPH, which exhibits low virulence on resistant rice varieties, is widely distributed across southeast Asia and primarily parasitizes susceptible varieties such as TN1 (Alam & Cohen, 1998). Rearing biotype 1 BPH on resistant rice variety YHY15 (carrying *Bph15*) for several years resulted in the development of highly-virulent biotype Y BPH, which are able to overcome resistance conferred by *Bph15* (Jing et al., 2011). Similarly, after force-feeding 40 generations of local BPH using resistant IR56 rice (carrying *Bph3*), the resulting BPH population (IR56-BPH) was able to overcome *Bph3*-conferred resistance (Zheng et al., 2016). In addition, the Mudgo BPH population has been reported to cause substantial damage to Mudgo rice plants (carrying *Bph1*) (Ji et al., 2013; Wan et al., 2019). In response to selective pressures imposed by these resistance genes, BPH populations accumulate adaptations over generations which eventually allow them to overcome such resistance mechanisms. In effect, this process constitutes a loss of resistance in formerly-resistant rice varieties against them. However, the effectiveness of resistance strategies used by different rice varieties against BPH populations with varying levels of virulence requires further investigation. Such combinations of avirulent/virulent BPH and resistant rice provide ideal models for studying resistance adaptation mechanisms.

Just as plants have evolved intricate defense mechanisms to protect themselves against herbivorous insects, insects have in turn developed strategies to overcome plant defenses (Liu Q. et al., 2021). Interactions between plants and insects involve an array of molecular, biochemical, and physiological processes occurring at multiple levels. Multi-omics analyses integrate genomics, transcriptomics, proteomics, and metabolomics, as well as other “omics” approaches, in order to clarify the intricate signaling pathways, molecular responses, and biochemical processes involved in the dynamic interplay between plants and insects (Wang et al., 2020; Shi et al., 2023). Transcriptional profiling has aided our understanding of the defense mechanisms utilized by rice against BPH. For example, research suggests that BPH infestation results in the upregulation of genes involved in signaling, oxidative stress, pathogen-related response, and macromolecule degradation, as well as the downregulation of genes associated with flavonoid biosynthesis, photosynthesis, and cell growth (Zhang et al., 2004; Yuan et al., 2005; Wang et al., 2008). In addition, microarray analyses of BPH-infested Rathu Heenati and TN1 rice underscore the importance of TFs and phytohormones in the defense response (Wang et al., 2012; Li et al., 2017).

MicroRNAs (miRNAs) are small (approximately 21 nt in length) regulatory RNAs produced through endonucleolytic processing of hairpin precursors (Axtell and Meyers, 2018). miRNAs regulate gene expression by binding to complementary sequences in mRNA molecules, resulting in degradation and/or translational inhibition (Bartel, 2009). In plants, miRNAs are involved in various processes such as phytohormone signaling; abiotic and biotic stress response (Zhang et al., 2013, 2016; Li et al., 2017; Natarajan et al., 2018; Salvador-Guirao et al., 2018; Zhang et al., 2018; Liu X. et al., 2021); and leaf, flower, shoot, root, and vascular tissue development (Marin et al., 2010; Peng et al., 2014; Mangrauthia et al., 2017). However, only a few miRNAs have been found to play roles in the insect-plant interaction (Jing et al., 2023). For example, in an investigation of resistant and susceptible rice varieties, the BPH-responsive miRNAs miR156 and miR396 were found to negatively regulate BPH resistance by regulating JA and flavonoid biosynthesis, respectively (Wu et al., 2017; Ge et al., 2018; Dai et al., 2019). Studies of BPH-responsive mRNA and miRNA transcriptomes have uncovered certain universal responses of rice to BPH infestation (Li et al., 2017; Wu et al., 2017; Nanda et al., 2020). Additionally, integrated expression profiling of miRNAs and target genes associated with the BPH-rice interaction has been conducted (Tan et al., 2020). Such combined miRNA and mRNA analyses will be key to unraveling the transcriptional responses of rice to BPH infestation.

In this study, we employed high-throughput sequencing to analyze the mRNA and miRNA expression profiles of YHY15 rice seedlings infested with either biotype 1 BPH (avirulent population) or biotype Y BPH (virulent population). Furthermore, we combined sequence analysis and physiological assays to reveal the underlying resistance mechanisms of rice against BPH. The findings presented in this work will provide a valuable resource for further genome-wide investigations of BPH-responsive genes, as well as studies of *Bph15*-mediated resistance. Moreover, these findings improve our understanding of the intricate interactions between rice and BPH, and may be used in the development of effective BPH management strategies.

## 2 Materials and methods

### 2.1 Plant and insect materials

In this study, we used two rice varieties (Taichung Native1 [TN1] and YHY15) and two BPH populations (biotype 1 and biotype Y). TN1 is a susceptible rice cultivar, while YHY15 is a recombinant inbred line (RIL) derived from the RI93 × TN1 F2 population carrying resistance gene *Bph15* (Yang et al., 2004). Biotype 1 BPH (avirulent) originated from Wuhan University (China) and are reared on TN1. Biotype Y BPH (virulent) were developed by rearing biotype 1 BPH on YHY15 plants beginning in January 2007 (Jing et al., 2011). All rice plants were grown from seeds sown in sponges (6 cm diameter, 2 cm height), with 8 rice plants per cup. Rice plants were reared in a controlled-environment incubator maintained at 30 ± 2 °C during daytime hours (16 h, 06:00–22:00) and 28 ± 2 °C during nighttime hours (8 h, 22:00–06:00). Rice plants were grown for approximately 2-5 weeks following sowing, depending on experimental needs. The BPH populations were reared at Xinyang Normal University (China) under the following conditions: 26 ± 1 °C, 16 h light/8 h dark cycle. Third instar BPH nymphs were used for the infestation experiments.

### 2.2 Evaluation of BPH resistance in rice

At the two-leaf (2-week-old) stage, YHY15 rice plants were infested with third instar biotype 1 or biotype Y BPH nymphs at a rate of 15 nymphs per seedling. The growth status of each plant was photographically recorded daily until all biotype Y-infested seedlings died. Each experiment consisted of three biological replicates.

### 2.3 Measurement of BPH weight gain and honeydew excretion

BPH weight gain and honeydew excretion were measured as described previously (Shi et al., 2021). Briefly, newly-emerged adult female BPH were weighed using an electronic balance (Mettler Toledo, MS105DU, Switzerland) and subsequently separated into pre-weighed parafilm sachets (2 × 2.5 cm) fixed to the leaf sheaths of 4-week-old rice plants. After 48 h, the insects were carefully removed from the sachets, and both the insect and the honeydew in each sachet were separately weighed. Weight gain was calculated by comparing each insect’s weight before and after feeding, and the weight gain ratio was calculated by dividing the weight gain by the initial weight. Both the weight gain and honeydew excretion assays were conducted using at least 37 replicates.

### 2.4 BPH infestation and sample collection

Three-week-old YHY15 rice seedlings were infested with third-instar biotype 1 (RT) and biotype Y (RY) BPH at a rate of 15 nymphs per seedling. Each treatment consisted of three biological replicates, with six seedlings per replicate. For RNA-seq and miRNA-seq analysis, leaf sheaths were collected from non-infested controls (0 h), and during early (6 h) and late (48 h) infestation. According to their time of collection, samples of non-infested rice plants were labeled ‘R0’, samples of biotype 1-infested rice plants were labeled as ‘RT6’ or ‘RT48’, and samples of biotype Y-infested rice plants were labeled as ‘RY6’ or ‘RY48’. Each sampled leaf sheath blade was excised, frozen in liquid nitrogen, and stored at -80 °C for further use.

### 2.5 mRNA transcriptome sequencing and analysis

#### 2.5.1 RNA extraction, quantification, and qualification

Total RNA was isolated from rice samples using RNA Trizol reagent (Life Technologies, NY, USA), following the manufacturer’s instructions. RNA degradation and contamination were evaluated using 1% agarose gels. RNA purity was quantified using a NanoPhotometer spectrophotometer (Implen, CA, USA). RNA integrity was assessed using an RNA Nano 6000 Assay Kit for the Bioanalyzer 2100 system (Agilent Technologies, CA, USA).

#### 2.5.2 Library construction, quality control, and sequencing

RNA libraries were generated using 1 µg of RNA per sample and the NEBNext Ultra RNA Library Prep Kit for Illumina. Index codes were incorporated to distinguish between samples. Briefly, mRNA was purified using poly-T oligo-attached magnetic beads, followed by fragmentation using divalent cations. First-strand cDNA synthesis was carried out using random hexamer primers and M-MuLV Reverse Transcriptase. The second strand was synthesized using DNA Polymerase I and RNase H. Overhangs were blunted using exonucleases or polymerases and NEBNext adaptors were ligated following adenylation. The library fragments (250~300 bp) were purified using an AMPure XP system. The USER Enzyme was applied to size-selected, adaptor-ligated cDNA prior to PCR. PCR was carried out using Phusion High-Fidelity DNA polymerase, Universal PCR primers, and Index (X) Primer. The PCR products were purified using an AMPure XP system, and library quality was evaluated using an Agilent Bioanalyzer 2100 system. Index-coded samples were clustered with a cBot Cluster Generation System using a TruSeq PE Cluster Kit v3-cBot-HS (Illumina), according to the manufacturer’s instructions. After clustering, the libraries were sequenced on an Illumina NovaSeq platform, generating 150 bp paired-end reads.

#### 2.5.3 Data analysis

Raw data (fastq format) were first processed using in-house perl scripts. In this step, low-quality reads, adapter sequences, and poly-Ns were removed. Subsequently, the Q20, Q30, and GC content of the clean reads were calculated. All downstream analyses utilized only clean, high-quality data. Next, the reference genome and gene model annotation files were downloaded. An index of the reference genome was constructed, and clean paired-end reads were aligned to the reference genome, using Hisat2 (v2.0.5). The mapped reads from each sample were assembled with StringTie (v1.3.3b) using a reference-based approach. featureCounts (v1.5.0-p3) was used to count the number of reads mapped to each gene. The Transcripts Per Kilobase Million (TPM) of each gene was quantified based on the gene length and the number of reads mapped to the gene. Differentially expressed genes (DEGs) were identified using the R (v1.16.1) package DESeq2, with three biological replicates per treatment, according to the following criteria: *P*-value < 0.05, false discovery rate (FDR) < 5, and absolute value of log_2_ fold change (FC) ≥ 1. The DEGs underwent additional screening through soft clustering using the Mfuzz package, employing a fuzzy c-means algorithm, as previously reported (Kumar and Futschik, 2007). DEGs exhibiting similar expression patterns were categorized into 20 clusters, and the genes within these clusters were subjected to Gene Ontology (GO) and Kyoto Encyclopedia of Genes and Genomes (KEGG) analyses. GO annotations were downloaded from NCBI (http://www.ncbi.nlm.nih.gov/) and GO (http://www.geneontology.org/). The KEGG database was used to identify BPH-responsive pathways. Fisher’s exact tests were applied to identify significant GO and KEGG categories according to the absolute values of *P* < 0.05 and FDR < 0.05.

### 2.6 miRNA transcriptome sequencing and analysis

#### 2.6.1 RNA extraction, quantification, and qualification

RNA extraction, quantification, and qualification were conducted as described in section 2.5.1.

#### 2.6.2 Library construction, quality control, and sequencing

Briefly, 3’ and 5’ adaptors were ligated to the 3’ and 5’ ends of small RNAs, respectively. Next, first strand cDNA was synthesized after hybridization with the reverse transcription primer. The double-stranded cDNA library was generated through PCR enrichment. After purification and size selection, libraries with 18~40 bp insertions were selected for Illumina sequencing with SE50. The library was quantified with Qubit and real-time PCR and the library size distribution was evaluated with a Bioanalyzer. Quantified libraries were pooled and sequenced on an Illumina platform, according to the effective library concentration and amount of data required.

#### 2.6.3 Data analysis

Raw data (fastq format) were first processed using in-house perl and python scripts. In this step, low-quality reads; reads containing poly-Ns, 5’ adapter sequences, or poly-As/Ts/Gs/Cs; and reads missing 3’ adapter sequences or insert tags were removed. Subsequently, the Q20, Q30, and GC content of the clean reads were calculated. All downstream analyses utilized only clean, high-quality data. Small RNA tags were mapped to the reference sequence using Bowtie (Langmead et al., 2009), either without mismatches or with only one mismatch, to analyze their expression and distribution. Mapped small RNA tags were used to identify known miRNAs, with miRBase (v22.0) used as a reference. miRNAs were identified using a modified version of mirdeep2 (Friedlander et al., 2012) and srna-tools-cli was used to draw the secondary structures. Custom scripts were used to obtain miRNA counts as well as to determine base bias at the first position of identified miRNAs of a certain length and at each position of all identified miRNAs. To remove tags originating from protein-coding genes, repeat sequences, rRNA, tRNA, snRNA, and snoRNA, small RNA tags were mapped to RepeatMasker, the Rfam database, or species-specific data. DEGs were identified using DESeq2. *P*-values were adjusted using the Benjamini & Hochberg method. A corrected *P*-value of 0.05 was selected as the threshold for determining significantly differential expression.

### 2.7 Analysis of transcriptional signatures of phytohormone responses

To identify the transcriptional signatures of BPH-responsive phytohormone responses, Hormonometer was used to compare gene expression in rice with gene expression in phytohormone-treated *Arabidopsis thaliana* (Volodarsky et al., 2009; Liu et al., 2016). Specifically, only orthologous genes detected in the RNA-seq analysis which were related to the *Arabidopsis thaliana* probe set identifiers were selected for further analyses.

### 2.8 qRT–PCR validation of DEGs

First-strand cDNA was synthesized using a PrimeScript RT Reagent Kit with gDNA Eraser (Takara, Japan), according to the manufacturer’s instructions. qRT-PCR assays of candidate genes were conducted using a PrimeScript™ RT reagent Kit with gDNA Eraser (Perfect Real Time) (Takara, RR047A, China). qRT-PCR was carried out on a CFX96 Real-Time System (Bio-Rad, CA, USA) according to the following protocol: 95 °C for 5 min, followed by 40 cycles at 95 °C for 5 s, 60 °C for 30 s, and 72 °C for 30 s. Relative gene expression was calculated with the 2−11Ct method, using *PP2A* as the reference gene. All primer sequences are listed in **Supplementary Table 1**.

### 2.9 Callose staining and evaluation

Callose staining and evaluation were performed as described in a previous study (Hu et al., 2017). Briefly, fresh sheaths collected from two-leaf stage rice seedlings infested with either biotype 1 or Y BPH for 48 h were fixed in an ethanol:acetic acid (3:1 v/v) solution for 5 h. The fixative was changed frequently to ensure thorough fixing and clearing. The samples were then rehydrated successively in 70% ethanol for 2 h, in 50% ethanol for 2 h, and in water overnight. After rinsing three times with water, the samples were treated with 10% NaOH for 1 h to make the tissues transparent. After rinsing four times with water, the samples were incubated in 150 mM K_2_HPO_4_ (pH 9.5) containing 0.01% aniline blue for 4 h. Finally, the samples were mounted on a slide and callose deposits were observed with a positive fluorescence microscope (Nikon, Eclipse 80i, Japan) under the UV channel.

### 2.10 Statistical analysis

Statistical analyses were conducted using R (v4.0.4) and SPSS (v22.0) (IBM SPSS, Somers, NY, USA). Two-sided Student’s *t*-tests were used to determine statistically significant differences between groups. All bioinformatics analyses were conducted using R packages.

## 3 Results

### 3.1 YHY15 exhibits differential resistance to biotype 1 and biotype Y BPH

**Figure 1:**
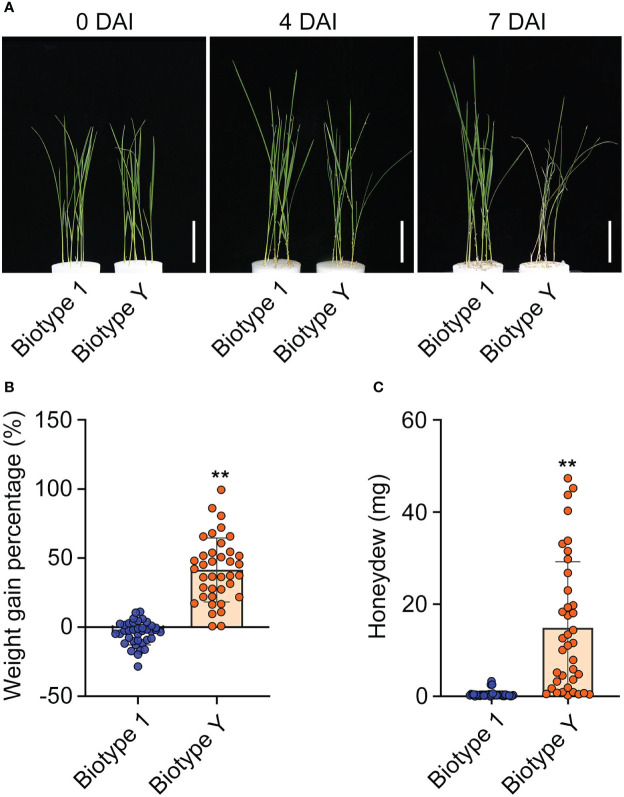
Resistance of YHY15 rice plants against biotype 1 and biotype Y BPH. **(A)** YHY15 seedlings infested with biotype 1 or biotype Y BPH. DAI, days after infestation. Scale bar = 5 cm. **(B)** BPH weight gain ratio on YHY15 seedlings. **(C)** BPH honeydew excretion on YHY15 seedlings. Data are shown as means ± SD of 38 biological replicates in **(B)** and **(C)**. Biotype 1, biotype 1 BPH-infested YHY15 seedlings; Biotype Y, biotype Y BPH-infested YHY15 seedlings. Asterisks indicate statistically significant differences between YY and TY (two-tailed Student’s *t*-test, **P < 0.01).

YHY15 rice, which contains the BPH resistance gene *Bph15*, exhibits robust resistance against avirulent BPH biotypes (i.e., biotype 1) (Yang et al., 2004). In this study, YHY15 seedlings were subjected to infestation by either biotype 1 or biotype Y BPH. The results suggest that YHY15 exhibited distinct responses under the two infestation scenarios. In response to infestation with biotype Y BPH, YHY15 seedlings exhibited signs of withering at 4 days and eventually wilted completely by 7 days. In contrast, seedlings infested with biotype 1 BPH remained healthy and continued to grow vigorously (**Figure 1A**). To evaluate the ability of YHY15 rice to affect the biology of BPH insects, we measured the weight gain and amount of honeydew produced by the two BPH populations. As expected, biotype Y exhibited a significantly higher weight gain ratio (mean = 41.4%) than biotype 1 (mean = −2.2%) (**Figure 1B**). In addition, biotype Y produced significantly more honeydew (mean = 16.49 mg) than biotype 1 (mean = 0.57 mg) (**Figure 1C**). These findings indicate that YHY15 rice plants are highly resistant to biotype 1 BPH but are susceptible to biotype Y BPH, as previously reported (Yang et al., 2004; Jing et al., 2011; Guan et al., 2022). Overall, these results provide strong evidence for the contrasting responses of YHY15 rice to BPH with different levels of virulence, and highlight the efficacy of the *Bph15* gene in conferring resistance against specific BPH strains.

### 3.2 Overview of the miRNA- and RNA-seq results

To better understand the mechanisms underlying the differential resistance displayed by YHY15 rice to virulent and avirulent BPH, we performed RNA and miRNA sequencing analyses using leaf sheaths from YHY15 seedlings infested with either biotype 1 (RT) or Y (RY) BPH for either 6 (RT6, RY6) or 48 h (RT48, RY48). Un-infested rice plants were used as controls and named as R0. After constructing and sequencing the mRNA/miRNA libraries, high-quality raw sequence reads were normalized and subjected to further analysis.

**Figure 2:**
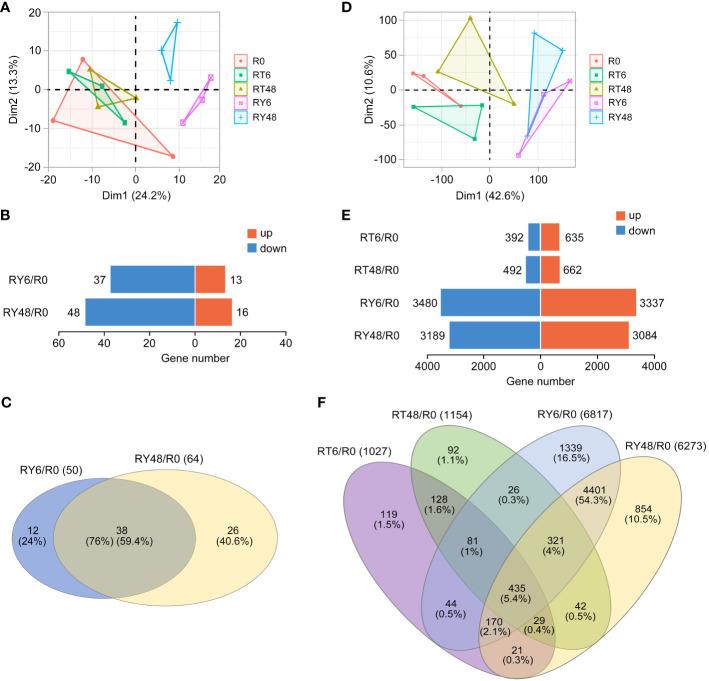
Overview of miRNA-seq and RNA-seq results. **(A)** Principal component analysis (PCA) of miRNA-seq data from five comparisons. **(B)** Number of miRNAs up- or downregulated in all comparisons (∣log_2_ fold change∣ > 1, *P* < 0.05). **(C)** Venn diagrams of differentially expressed miRNAs in all comparisons. **(D)** PCA of RNA-seq data from five comparisons. **(E)** Number of mRNAs up- or downregulated in all comparisons (|log_2_ fold change| > 1, *P* < 0.05). **(F)** Venn diagrams of differentially expressed mRNAs in all comparisons. There are four comparisons: RT6/R0, RT48/R0, RY6/R0, RY48/R0. R0, uninfected controls; RT6, YHY15 seedlings infested with biotype 1 BPH for 6 h; RT48, YHY15 seedlings infested with biotype 1 BPH for 48 h; RY6, YHY15 seedlings infested with biotype Y BPH for 6 h; RY48, YHY15 seedlings infested with biotype Y BPH for 48 h.

Out of the 738 identified miRNAs, miR396, miR167, miR166, miR162, miR159, miR156, miR820, miR408, miR1425, miR1862, miR444, and miR827 exhibited the highest relative abundance (**Supplementary Table 2**). Principal component analysis (PCA) of the miRNA-seq data revealed significant variation between the R0 group and the RY6 or RY48 group, indicating that biotype Y BPH infestation led to distinct fluctuations in the miRNA profiles (**Figure 2A**). Among the 50 differentially expressed miRNAs (DEMs) detected in R0 *vs* RY6, 13 were upregulated and 37 were downregulated (**Figure 2B**; **Supplementary Table 3**). As infection progressed, more DEMs (64) were identified in R0 *vs* RY48, 16 of which were upregulated and 48 were downregulated (**Figure 2B**; **Supplementary Table 3**). In total, 38 DEMs were shared between R0 *vs* RY6 and R0 *vs* RY48, with 12 unique DEMs detected at the earlier time point and 26 at the later time point (**Figure 2C**). These results suggest that many more miRNAs are involved in the interaction between YHY15 and biotype Y BPH than in the interaction between YHY15 and biotype 1 BPH. Among these, miR156, miR5076, miR1856, miR398, miR5072, miR5079, miR408, miR2873, and miR169 exhibited significant variation in amplitude (**Table 1**). Many of these miRNAs have been reported to play specific roles in plant developmental processes and biotic and abiotic stress responses (Sharma et al., 2015; Zhao et al., 2017; Lin et al., 2018; Liu et al., 2019; Liebsch and Palatnik, 2020; Li et al., 2020; Liu X. et al., 2021; Gao et al., 2022; Pachamuthu and Hari Sundar, 2022; Zhao et al., 2022). Their high abundance and variable expression patterns suggest that they may contribute to BPH resistance in YHY15 seedlings, and therefore subsequent analyses were conducted on these miRNAs.

**Table 1:** Candidate BPH resistance-related DEMs.

osa-miRNA	Fold change (log_2_)		Target genes
	RY6/R0	RY48/R0	
miR11342-3p	1.22	–	LOC107277366; LOC107278339; LOC9270471; LOC4347355; LOC4327594
miR1320-3p	–	-4.28	LOC4341008; LOC4341009; LOC9272503; LOC107276137; LOC112937314
miR1423-3p	–	1.18	LOC4334792; LOC4334793; LOC4352758; LOC4352759; LOC112936210
miR1425-3p	1.71	–	LOC107277584; LOC4347752; LOC4347753; LOC9267694; LOC9267104
miR1432-5p	-1.59	-1.28	LOC4331372; LOC9270922; LOC107281270; LOC112939644; LOC9269030
miR156a	-1.28	-1.56	LOC4338174; LOC4333935; LOC4333937; LOC4328870; LOC4332289
miR156b-5p	-1.28	-1.56	LOC4338174; LOC4333935; LOC4333937; LOC4328870; LOC4332289
miR156c-3p	–	-4.39	LOC4335110; LOC4335111; LOC9268400; LOC107278252; LOC107279186
miR156c-5p	-1.28	-1.56	LOC4338174; LOC4333935; LOC4333937; LOC4328870; LOC4332289
miR156d	-1.02	-1.08	LOC4338174; LOC4333935; LOC4333937; LOC4328870; LOC4332289
miR156e	-1.28	-1.56	LOC4338174; LOC4333935; LOC4333937; LOC4328870; LOC4332289
miR156f-3p	–	-3.36	LOC9269030; LOC4348312; LOC4332049; LOC9269785; LOC4335110
miR156f-5p	-1.02	-1.08	LOC4338174; LOC4333935; LOC4333937; LOC4328870; LOC4332289
miR156g-3p	–	-4.39	LOC4335110; LOC4335111; LOC9268400; LOC107278252; LOC107279186
miR156g-5p	-1.28	-1.56	LOC4338174; LOC4333935; LOC4333937; LOC4328870; LOC4332289
miR156h-3p	–	-3.36	LOC9269030; LOC4348312; LOC4332049; LOC9269785; LOC4335110
miR156h-5p	-1.02	-1.08	LOC4338174; LOC4333935; LOC4333937; LOC4328870; LOC4332289
miR156i	-1.28	-1.56	LOC4338174; LOC4333935; LOC4333937; LOC4328870; LOC4332289
miR156j-5p	-1.02	-1.08	LOC4338174; LOC4333935; LOC4333937; LOC4328870; LOC4332289
miR156k	–	-2.01	LOC4331703; LOC4338174; LOC4335101; LOC107276848; LOC4341195
miR156l-3p	–	-3.36	LOC9269030; LOC4348312; LOC4332049; LOC9269785; LOC4335110
miR166a-5p	1.53	–	LOC4326513; LOC107277945; LOC107277317; LOC4347823; LOC4343486
miR166c-5p	–	-1.13	LOC4347823; LOC107277945; LOC4326513; LOC112939883; LOC4343038
miR166d-5p	-1.47	-1.97	LOC4326513; LOC4343486; LOC9266571; LOC9268304; LOC4338511
miR166e-5p	1.94	–	LOC4326513; LOC107277945; LOC107277317; LOC4347823; LOC4343486
miR166j-5p	-1.75	-1.81	LOC9269030; LOC107281130; LOC4332497; LOC4325456; LOC4325457
miR166k-3p	–	1.18	LOC107281270; LOC4343122; LOC4328998; LOC107277945; LOC4326262
miR166l-3p	–	1.17	LOC107281270; LOC4343122; LOC4328998; LOC107277945; LOC4326262
miR167h-3p	-1.85	-2.33	LOC107278090; LOC107279103; LOC9267730; LOC4348767; LOC112935985
miR169h	–	-2.37	LOC107277945; LOC107277048; LOC4340902; LOC107277584; LOC4347752
miR169i-3p	–	-1.11	LOC9269693; LOC4352155; LOC4352156; LOC107277945; LOC112938521
miR169i-5p.1	–	-2.37	LOC107277945; LOC107277048; LOC4340902; LOC107277584; LOC4347752
miR169j	–	-2.37	LOC107277945; LOC107277048; LOC4340902; LOC107277584; LOC4347752
miR169k	–	-2.37	LOC107277945; LOC107277048; LOC4340902; LOC107277584; LOC4347752
miR169l	–	-2.37	LOC107277945; LOC107277048; LOC4340902; LOC107277584; LOC4347752
miR169r-3p	–	-2.40	LOC9269030; LOC4330994; LOC4330995; LOC107279587; LOC107277598
miR171a	1.40	1.45	LOC4331702; LOC107276230; LOC107276994; LOC4351951; LOC4349818
miR1851	-1.27	–	LOC4342932; LOC9270958; LOC4342934; LOC4335125; LOC4334367
miR1856	-3.38	-3.56	LOC4345309; LOC4345310; LOC4325535; LOC4325537; LOC107275864
miR1861c	1.22	–	LOC4331658; LOC112938679; LOC107277945; LOC9267779; LOC4330644
miR1874-3p	1.34	–	LOC107277945; LOC4346563; LOC4346564; LOC4350472; LOC107276848
miR2121a	–	1.14	LOC112937314; LOC9266659; LOC4340263; LOC112938789; LOC107276637
miR2121b	–	1.14	LOC112937314; LOC9266659; LOC4340263; LOC112938789; LOC107276637
miR2871a-5p	-1.33	-1.39	LOC4331613; LOC4331617; LOC4350261; LOC112936180; LOC4330291
miR2873a	-2.05	-2.58	LOC4326290; LOC107277945; LOC9268610; LOC4347267; LOC9268583
miR319a-3p	5.40	4.59	LOC4347750; LOC107276213; LOC4347751; LOC9271092; LOC4347551
miR319a-3p.2-3p	4.05	4.58	LOC4337861; LOC4337862; LOC4326585; LOC107276637; LOC4335012
miR319b	3.88	4.42	LOC4337861; LOC4337862; LOC4326585; LOC107276637; LOC4335012
miR396a-3p	-1.05	-1.34	LOC4337237; LOC9268610; LOC4331423; LOC4326513; LOC4346184
miR396c-5p	1.01	1.25	LOC112938789; LOC4345308; LOC4331372; LOC9270922; LOC107277366
miR397a	-1.69	-1.89	LOC9267104; LOC4329608; LOC4339828; LOC4326290; LOC107277317
miR397b	-1.73	-1.66	LOC107277945; LOC9270668; LOC107276994; LOC4351951; LOC107277567
miR3980a-5p	-1.09	-1.24	LOC4352509; LOC4337053; LOC4349876; LOC112937650; LOC4352872
miR3980b-5p	-1.09	-1.24	LOC4352509; LOC4337053; LOC4349876; LOC112937650; LOC4352872
miR398a	-4.42	–	LOC4331702; LOC4335368; LOC4349919; LOC9268603; LOC4324333
miR398b	-2.56	-2.33	LOC4331702; LOC4335368; LOC4335110; LOC4335111; LOC4349919
miR399i	-1.30	–	LOC107277584; LOC4347752; LOC4347753; LOC9267694; LOC4348598
miR408-3p	-1.81	-1.57	LOC107276637; LOC4335125; LOC107277770; LOC9267065; LOC112936987
miR408-5p	-2.28	-2.45	LOC4335589; LOC9272252; LOC4334900; LOC4346674; LOC4329468
miR444a-3p.1	–	-1.83	LOC107281435; LOC4335110; LOC4335111; LOC9266659; LOC4340263
miR444b.1	-1.04	–	LOC9269785; LOC9272338; LOC107277945; LOC107280439; LOC4331618
miR444c.1	-1.04	–	LOC9269785; LOC9272338; LOC107277945; LOC107280439; LOC4331618
miR444d.1	–	-1.83	LOC107281435; LOC4335110; LOC4335111; LOC9266659; LOC4340263
miR5072	-2.47	-1.86	LOC107277317; LOC9266659; LOC4340263; LOC9272689; LOC112936211
miR5076	-3.33	-2.65	LOC107277945; LOC107279298; LOC4350353; LOC107275975; LOC4351913
miR5079a	-2.38	-2.66	LOC9270668; LOC4326380; LOC4347267; LOC107277945; LOC4332858
miR5079b	-2.38	-2.66	LOC9270668; LOC4326380; LOC4347267; LOC107277945; LOC4332858
miR528-5p	-1.58	-1.31	LOC4347809; LOC4335364; LOC107276241; LOC112936210; LOC107279401
miR535-5p	-1.06	–	LOC4324213; LOC112937314; LOC107277945; LOC107276994; LOC4351951
miR5505	2.48	1.77	LOC4350473; LOC4352601; LOC4352606; LOC4331386; LOC4338511
miR5801b	1.07	1.34	LOC9266659; LOC4340263; LOC107282017; LOC9268610; LOC112936211
miR5816	–	1.41	LOC107279289;LOC107275804;LOC107278811;LOC9269030;LOC112937448
miR818b	–	1.14	LOC4329368; LOC107277317; LOC4335309; LOC4329911; LOC107275634
miR818d	–	1.06	LOC4329368; LOC107277317; LOC4335309; LOC4329911; LOC107275634
miR818e	–	1.14	LOC4329368; LOC107277317; LOC4335309; LOC4329911; LOC107275634
miR827	-1.06	-1.12	LOC4345821; LOC4350156; LOC4352108; LOC4335589; LOC9272252

In comparison, PCA of the RNA-seq data revealed distinct separation among all groups (**Figure 2D**; **Supplementary Table 4**). Through comparison of RNA expression levels across different groups, we identified 1027 and 1154 differential expression analysis (DEGs) in R0 *vs* RT6 and R0 *vs* RT48, respectively. Notably, a larger number of DEGs were observed in R0 *vs* RY6 and R0 *vs* RY48 (6817 and 6273, respectively), suggesting that infestation with biotype Y BPH results in more severe effects than infestation with biotype 1 BPH (**Figure 2E**). A Venn diagram was used to compare the expression patterns of DEGs in avirulent/virulent BPH-infested rice at the early (6 h) and late (48 h) stages of infestation, and the identified DEGs were subjected to GO and KEGG enrichment analyses (**Figure 2F**; **Supplementary Figure 1**; **Supplementary Table 5**). We identified 730 overlapping DEGs in R0 *vs* RT6 and R0 *vs* RY6 at the early infestation stage, which were enriched in cytochrome P450 (KEGG), protein modification-related biological processes (BP, GO), extracellular region (CC, GO), and monooxygenase activity (MF, GO) (**Figure 2F**; **Supplementary Figures 1A, B**). Specifically, genes in R0 *vs* RY6 were enriched in ribosome, photosynthesis proteins, carbon fixation in photosynthetic organisms, and DNA replication proteins (**Figure 2F**; **Supplementary Figures 2A, B**). However, genes in R0 *vs* RT6 did not exhibit these enrichments. We identified 827 overlapping DEGs in R0 *vs* RT48 and R0 *vs* RY48 at the late infestation stage, which were enriched in photosynthesis proteins, porphyrin metabolism, zeatin biosynthesis, and cytochrome P450, suggesting disturbance to the photosynthetic system (KEGG) (**Figure 2F**; **Supplementary Figures 3A, B**). Additionally, genes in R0 *vs* RY48 were enriched in ribosome-related pathways, DNA replication proteins, photosynthesis proteins, and carbon fixation in photosynthetic organisms (**Figure 2F**; **Supplementary Figures 4A, B**). These results indicate that infestation with biotype Y BPH results in damage to the photosynthetic system earlier than infestation with biotype 1 BPH, which is consistent with the phenotypic observations (**Figure 1A**).

### 3.3 Transcriptional responses in YHY15 rice to BPH infestation

**Figure 3:**
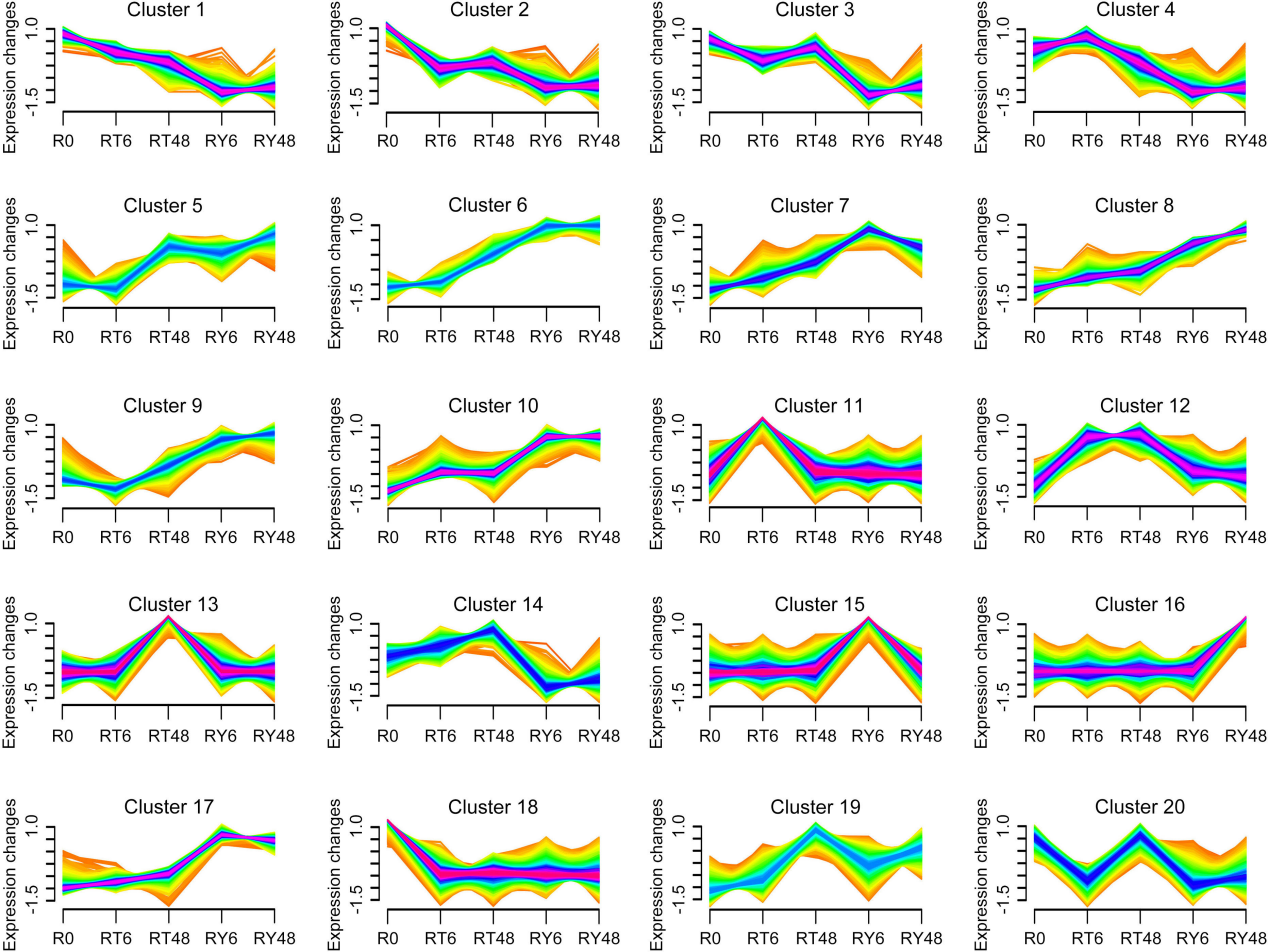
Clustering and time-course expression of genes after BPH infestation. The 20 distinct temporal gene expression patterns were computed with Mfuzz. The x axis represents the five treatment groups, and the y axis represents log_2_-transformed normalized intensity ratios for each group. R0, uninfected controls; RT6, YHY15 seedlings infested with biotype 1 BPH for 6 h; RT48, YHY15 seedlings infested with biotype 1 BPH for 48 h; RY6, YHY15 seedlings infested with biotype Y BPH for 6 h; RY48, YHY15 seedlings infested with biotype Y BPH for 48 h.

**Figure 4:**
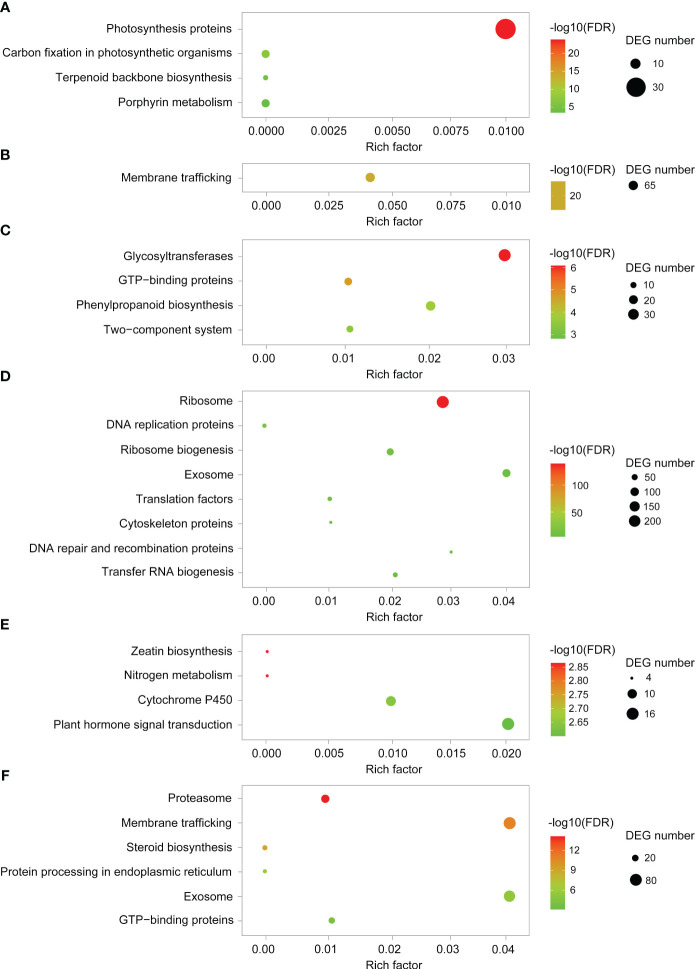
KEGG pathway enrichment analyses of representative clusters. **(A–F)** KEGG pathway enrichment analyses of cluster 1, 6, 7, 10, 11, and 17, respectively.

To elucidate the genome-wide responses of YHY15 rice to BPH infestation, the transcriptome was analyzed over the entire infection time course. According to Mfuzz analysis, distinct temporal patterns were clustered into 20 groups (**Figure 3**; **Supplementary Table 6**). Notably, clusters 1 to 4 exhibited significantly lower levels of transcription in RY6/48 compared to R0, whereas this trend was less pronounced in RT6/48 (**Figure 3**; **Supplementary Table 6**). The genes in these clusters were subjected to GO and KEGG enrichment analyses to explore their functions. Genes in cluster 1 were enriched in photosynthesis proteins, carbon fixation in photosynthetic organisms, terpenoid backbone biosynthesis, and porphyrin metabolism (**Figure 4A**; **Supplementary Table 7**). Accordingly, GO analysis confirmed that many of these genes were related to the photosynthesis biological process, as well as photosynthetic cellular components such as thylakoid, photosynthetic membrane, and photosystem (**Supplementary Figure 5A**; **Supplementary Table 7**). These results suggest that infestation with biotype Y BPH results in significantly greater physical impairment than does infestation with biotype 1 BPH, which was consistent with the growth observations (**Figure 1A**).

In clusters 5 to 10, gene expression levels increased significantly in the RY groups, but only slightly in the RT groups (**Figure 3**; **Supplementary Table 6**). Genes in cluster 6 (**Figure 4B**; **Supplementary Figure 5B**; **Supplementary Table 7**) and cluster 9 (data not shown) were enriched in membrane trafficking (KEGG), cell membrane systems (GO), and protein transporter (GO), suggesting that defense against BPH relies on membrane transport systems and cell secretion. In cluster 7, genes were enriched in glycosyltransferases, GTP-binding proteins, phenylpropanoid biosynthesis, and two-component system (**Figure 4C**; **Supplementary Figure 5C**; **Supplementary Table 7**). The phenylpropanoid pathway is involved in the production of various metabolites, including flavonoids, lignin, lignans, and cinnamic acid amide, among others (Dong and Lin, 2021). Genes in cluster 10 were enriched in ribosomes, DNA replication proteins, translation factors, and other transcriptional activity-related factors (**Figure 4D**; **Supplementary Figure 5D**; **Supplementary Table 7**). Overall, the transcriptional system, transmembrane transport system, exosome, and phenylpropanoid pathway are notably stimulated by BPH infestation, particularly infestation with biotype Y BPH, suggesting that they may be crucial for rice resistance to insects.

The expression patterns of several clusters appeared to be desynchronized, indicative of different responses to infestation by either biotype 1 or biotype Y BPH. In clusters 11 to 14, gene expression was increased at the early (6 h) and late (48 h) stages during infestation with biotype 1 BPH, but remained relatively stable during infestation with biotype Y BPH (**Figure 3**; **Supplementary Table 6**). These clusters were mainly enriched in zeatin biosynthesis, nitrogen metabolism, cytochrome P450, and plant hormone signal transduction (**Figure 4E**; **Supplementary Figure 5E**; **Supplementary Table 7**). In plants, cytochrome P450 catalyzes various primary and secondary metabolic reactions and is involved in the synthesis and metabolism of terpenoids, alkaloids, sterols, fatty acids, plant hormones, signaling molecules, pigments, flavonoids, and isoflavones, among others (Hansen et al., 2021). Such multifunctionality makes cytochrome P450 important in the plant defense against pests, diseases, and abiotic stressors. In addition, zeatin is the primary active component of the phytohormone CK. KEGG analysis revealed that the cytochrome P450-phytohormone signal transduction pathway was activated in YHY15 rice under biotype 1 BPH infestation. In contrast, gene expression in clusters 15 to 17 was increased at the early (6 h) and late (48 h) stages during infestation with biotype Y BPH (**Figure 3**; **Supplementary Table 6**). These genes were enriched in proteasome, membrane trafficking, steroid biosynthesis, protein processing in endoplasmic reticulum, exosome, and GTP-binding proteins (**Figure 4F**; **Supplementary Figure 5F**; **Supplementary Table 7**). These results suggest that cell secretion and extracellular materials are crucial for defense against insects. Finally, clusters 18 to 20 did not exhibit any notable tendencies (**Figure 3**).

### 3.4 Phytohormonal responses in YHY15 rice to BPH infestation

**Figure 5:**
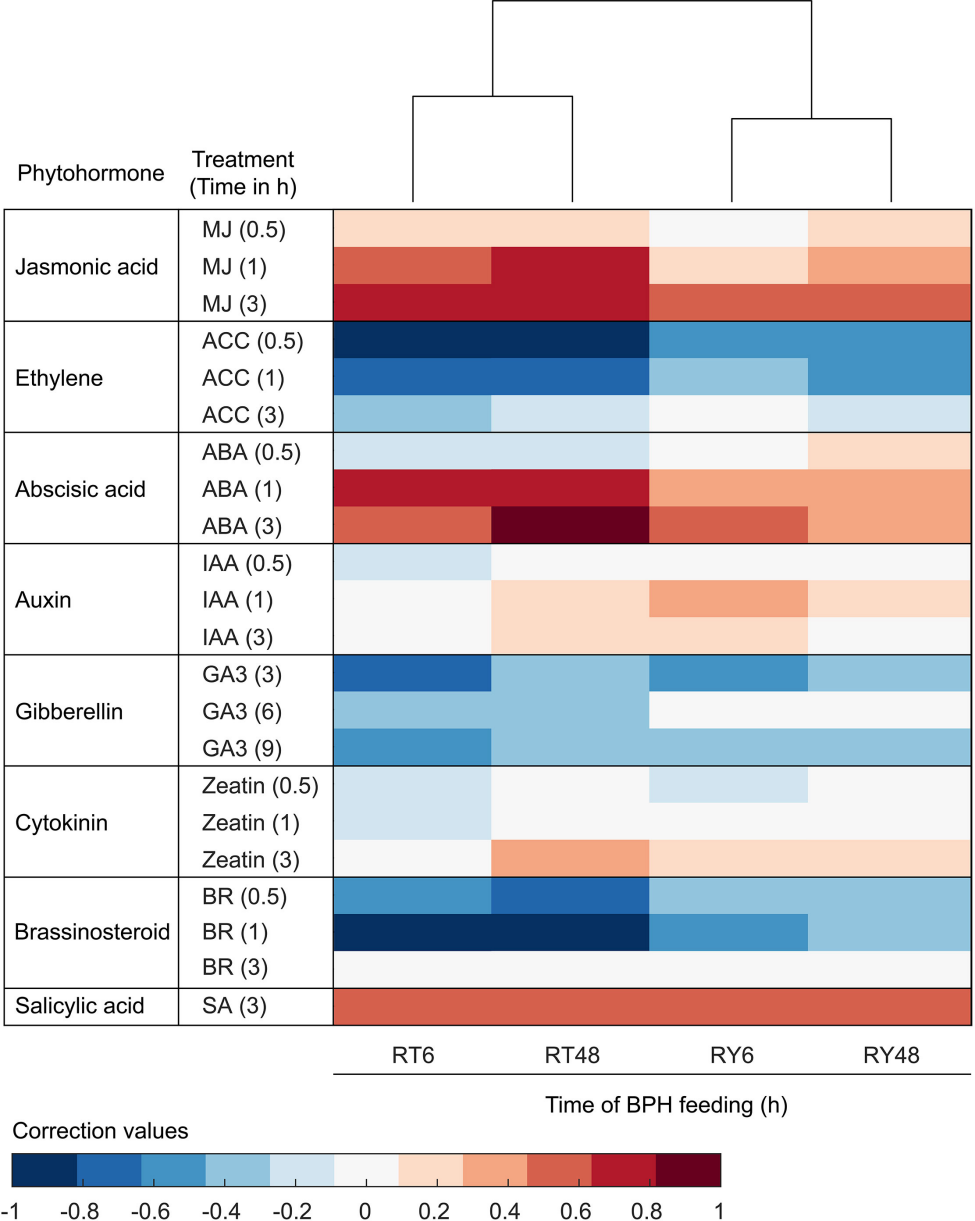
Comparison of transcriptomic phytohormone signatures between BPH-infested rice and phytohormone-treated *Arabidopsis thaliana*. Red shading indicates positive correlations and blue shading indicates negative correlations between BPH-infested rice and phytohormone-treated *A. thaliana*. MJ, methyl jasmonate; ACC, 1-aminocyclopropane-1-caroxylic acid (precursor of ethylene); ABA, abscisic acid; IAA, indole-3-acetic acid; GA3, gibberellic acid 3; ZT, zeatin; BR, brassinosteroid; SA, salicylic acid. R0, uninfected controls; RT6, YHY15 seedlings infested with biotype 1 BPH for 6 h; RT48, YHY15 seedlings infested with biotype 1 BPH for 48 h; RY6, YHY15 seedlings infested with biotype Y BPH for 6 h; RY48, YHY15 seedlings infested with biotype Y BPH for 48 h.

Both the clustering analysis and KEGG/GO enrichment analysis indicated that phytohormone signaling was differentially affected by infestation with either biotype 1 or Y BPH (**Figures 3**, **4E**). Consequently, we compared the expression of BPH-responsive genes in rice with those induced by exogenous phytohormone application in *A. thaliana*. According to Hormonometer analysis, a total of 15,603 *A. thaliana* orthologs of rice genes were selected for comparison (**Supplementary Table 8**). The results revealed that genes associated with JA-dependent signaling were significantly altered by infestation with biotype 1 BPH, but only moderately changed by infestation with biotype Y BPH (**Figure 5**). Genes associated with ABA exhibited a similar pattern, except for being negatively correlated with genes whose expression in *A. thaliana* was elicited 0.5 h after ABA treatment (**Figure 5**). In contrast, the expression of genes involved in the ET and BR pathways exhibited negative correlations with those responding to phytohormone application in *A. thaliana*, although with more moderate suppression in biotype Y BPH-infested rice (**Figure 5**). These results show that genes associated with the JA, ABA, ET, and BR pathways are the most responsive to BPH infestation, particularly in the case of biotype 1 BPH, indicating their crucial roles in the rice defense against insects.

**Figure 6:**
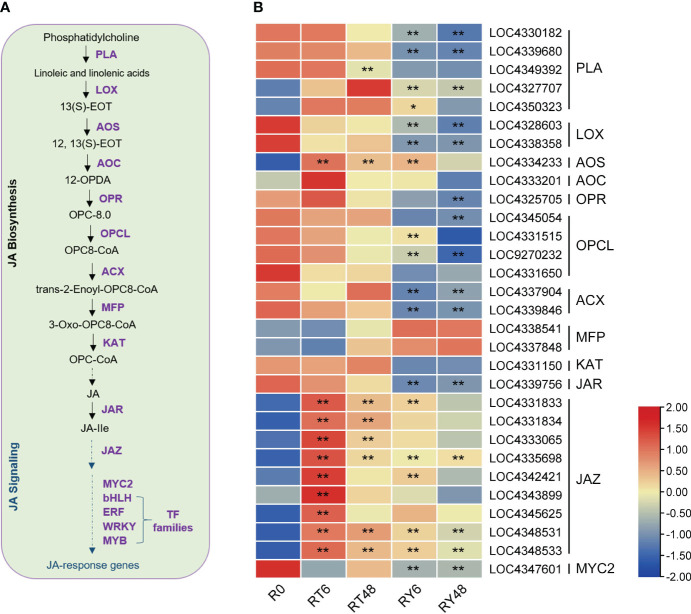
BPH-induced responses in the jasmonic acid (JA) pathway. **(A)** Overview of the JA pathway. **(B)** Heat map of the expression of genes associated with the JA pathway. Asterisks indicate statistically significant differences in gene expression at different time points in either biotype 1 or biotype Y BPH-infested rice relative to control (0 h) (*padj < 0.05, **padj < 0.01, via the Benjamini and Hochberg adjustment method). PLA, phospholipase A1; LOX, lipoxygenase; AOS, allene oxide synthase; AOC, allene oxide cyclase; OPR, 12-oxophytodienoate reductase; OPCL, OPC8-CoA ligase; ACX, acyl-CoA oxidase; MFP, multifunctional protein; KAT, 3-ketoacyl-CoA thiolase; JAR, jasmonate resistant; JAZ, jasmonate-ZIM domain. Transcription factor families: myelocytomatosis protein 2 (MYC2), basic helix-loop-helix (bHLH), ethylene responsive factor (ERF), WRKYGOK (WRKY), MYB. R0, uninfected controls; RT6, YHY15 seedlings infested with biotype 1 BPH for 6 h; RT48, YHY15 seedlings infested with biotype 1 BPH for 48 h; RY6, YHY15 seedlings infested with biotype Y BPH for 6 h; RY48, YHY15 seedlings infested with biotype Y BPH for 48 h.

Previous research suggests that JA is involved in BPH resistance (Xu et al., 2021). We found that the JA-dependent pathway was differentially induced by infestation with either biotype 1 or Y BPH, implying that JA plays a crucial role in rice defense (**Figure 5**). We further analyzed the expression of genes involved in the JA pathway, and found that most of the genes involved in the biosynthesis of JA and jasmonoyl-isoleucine (JA-Ile) (e.g., *LOX* and *JAR*) were significantly suppressed by BPH infestation, especially infestation with biotype Y BPH (**Figures 6A, B**). Accordingly, most JAZs, which inhibit JA signaling, were induced in RT6/48 and RY6/48 (**Figures 6A, B**). In addition, MYC2 was downregulated in YHY15 rice infested with biotype Y BPH, suggesting that the JA-dependent pathway was inactivated upon infestation with the virulent BPH (Ge et al., 2018). Finally, the expression of many TFs was altered in BPH-infested rice, especially rice infested with biotype Y BPH, including those belonging to the JA-responsive bHLH, ERF, WRKY, and MYB families (**Supplementary Figure 6**). These distinct response patterns imply that these TFs have functions in BPH defense, although further research is required to verify these functions. The expression profiles of the identified TFs can be found in **Supplementary Table 9**.

### 3.5 Integrated miRNA and mRNA transcriptional analyses

The functions of the identified miRNAs were initially evaluated by scanning the literature. It has been reported that miR156 negatively regulates BPH resistance in rice by altering the expression of genes related to JA biosynthesis and signaling (Ge et al., 2018). We observed that miR156 expression was significantly decreased in biotype Y BPH-infested rice. Consequently, WRKY53, several MPKs (JA biosynthesis repressors), and several JAZs (negative regulators of JA signaling) were upregulated (**Table 2**). These transcriptional alterations resulted in low JA expression and improved BPH resistance. According to other research, miR396 suppresses BPH resistance through the miR396b–growth regulating factor 8 (GRF8)–flavanone 3-dioxygenase (F3H)–flavonoid pathway (Dai et al., 2019). Therefore, we examined the expression of *miR396*, *GRF8*, and *F3H*. Mature OsmiR396a and OsmiR396b share the same sequence (Dai et al., 2019), and we observed that miR396a was downregulated by infestation with biotype Y BPH. In contrast, *GRF8* and *F3H*-*1* expression increased in RY6 and RY48. Notably, *GRF1* and two *flavanone 3-dioxygenase 2-like* genes exhibited the same trend (**Table 2**). These results suggest that the miR396b–GRF8–F3H–flavonoid pathway was activated in biotype Y BPH- infested rice. Genetic experiments indicate that miR398b negatively regulates pathogen-associated molecular pattern (PAMP)-induced callose deposition (Li et al., 2010). As miR398 expression was significantly downregulated in RY6/48, we further analyzed the transcription of callose deposition-related genes. Surprisingly, a callose synthase gene was activated following biotype Y BPH infestation, while a series of callose degradation genes were suppressed (**Table 2**). These results suggest that infestation with biotype Y BPH may lead to callose deposition in rice. Taken together, it appears that the expression of these miRNAs and their regulation of target genes may contribute the ability of YHY15 rice to resist infestation with biotype Y BPH.

**Table 2:** The miRNA-mRNA interactions related to plant resistance.

osa-miRNA	Gene ID	Fold change (log_2_)		Discription
		RY6/R0	RY48/R0	
miR156	LOC4338474	1.01	–	Probable WRKY transcription factor 26, WRKY53
	LOC4342017	1.23	1.08	Mitogen-activated protein kinase 12, MAP kinase 12
	LOC4339697	1.03	1.02	Mitogen-activated protein kinase 17 isoform X1, MAP kinase 17
	LOC4331834	2.40	1.64	Jasmonate ZIM domain-containing protein 10, OsJAZ10; protein TIFY 11b
	LOC4331833	2.37	–	Jasmonate ZIM domain-containing protein 11, OsJAZ11; protein TIFY 11c
	LOC4348533	2.76	2.11	Jasmonate ZIM domain-containing protein 12, OsJAZ12; protein TIFY 11d
	LOC4348531	5.83	4.65	Jasmonate ZIM domain-containing protein 13, OsJAZ13; protein TIFY 11e
	LOC4342421	4.59	–	Jasmonate ZIM domain-containing protein 2, OsJAZ2; protein TIFY 5
miR396	LOC4330903	2.99	3.06	Growth-regulating factor 1, OsGRF1
	LOC4350711	1.23	1.35	Growth-regulating factor 8, OsGRF8
	LOC9270463	4.59	4.02	Flavanone 3-dioxygenase 1, OsF3H-1
	LOC4345848	–	1.55	Flavanone 3-dioxygenase 2-like
	LOC4347916	2.56	2.20	Flavanone 3-dioxygenase 3-like
miR398	LOC4345025	-2.62	-2.00	endo-1,3;1,4-beta-D-glucanase
	LOC4338721	-2.84	-3.28	endo-1,3;1,4-beta-D-glucanase
	LOC4350272	-2.44	-1.40	endo-1,3;1,4-beta-D-glucanase
	LOC4350269	-2.48	-1.79	endo-1,3;1,4-beta-D-glucanase
	LOC4350270	-1.23	-1.26	endo-1,3;1,4-beta-D-glucanase
	LOC4345024	-2.17	-1.27	endo-1,3;1,4-beta-D-glucanase
	LOC9268304	–	-1.26	glucan endo-1,3-beta-glucosidase 12, putative beta-1,3-glucanase
	LOC4345052	–	-1.34	glucan endo-1,3-beta-glucosidase 7, putative beta-1,3-glucanase precursor
	LOC4326519	–	-2.13	glucan endo-1,3-beta-glucosidase, putative beta-1,3-glucanase precursor
	LOC4326518	-1.30	-1.59	glucan endo-1,3-beta-glucosidase, beta 1,3-glucanase
	LOC4338611	-1.58	-1.74	putative beta-1,3-glucanase
	LOC4334765	-3.09	–	probable glucan endo-1,3-beta-glucosidase A6, putative beta-1,3-glucanase
	LOC4339201	-1.79	-1.57	putative glucan endo-1,3-beta-glucosidase GVI, putative beta-1,3-glucanase
	LOC4327203	3.03	2.93	glucan endo-1,3-beta-glucosidase 13, putative elicitor inducible beta-1,3-glucanase NtEIG-E76
	LOC4332097	2.84	2.73	glucan endo-1,3-beta-glucosidase 3 isoform X1, Putative beta-1,3-glucanase
	LOC4334391	1.48	1.33	glucan endo-1,3-beta-glucosidase, putative beta-1,3 glucanase
	LOC4346925	–	–	probable endo-1,3(4)-beta-glucanase ARB_01444
	LOC4342136	1.41	1.40	callose synthase 3-like
	LOC4331485	-1.67	-1.36	callose synthase 3

### 3.6 Callose deposition is activated by BPH infestation

**Figure 7:**
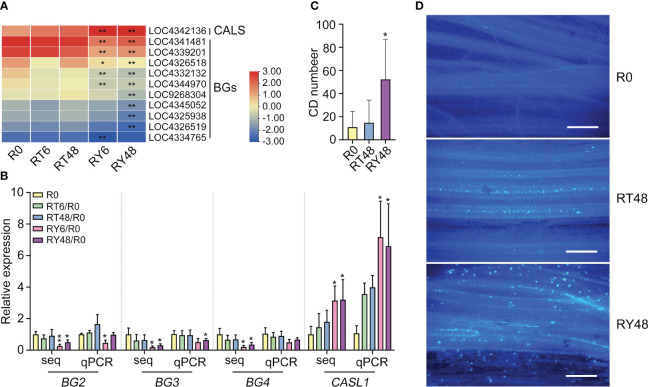
Induction of callose deposition in YHY15 rice plants following BPH infestation. **(A)** Heat map of the expression of genes associated with callose deposition and degradation. Asterisks indicate statistically significant differences in gene expression at different time points in either biotype 1 or biotype Y BPH-infested rice relative to control (0 h) (*padj < 0.05, **padj < 0.01, via the Benjamini and Hochberg adjustment method). **(B)** Expression patterns of callose deposition-related genes in BPH-infested rice plants. *UBQ* was used as a control. **(C)** Callose deposition (CD) in BPH-infested YHY15 plants. The images were taken 48 h after BPH infestation. Samples were collected from the outermost sheath. Callose deposition values are the means of 20 biological replicates. Asterisks indicate statistically significant differences between BPH-infested and uninfested rice plants (one-way ANOVA, *P < 0.05). **(D)** Callose deposition in YHY15 plants infested with either biotype 1 or biotype Y BPH. Scale bar = 20 μm. The images were taken 48 h after BPH infestation. Uninfested YHY15 plants in the same condition served as control. R0, uninfected controls; RT6, YHY15 seedlings infested with biotype 1 BPH for 6 h; RT48, YHY15 seedlings infested with biotype 1 BPH for 48 h; RY6, YHY15 seedlings infested with biotype Y BPH for 6 h; RY48, YHY15 seedlings infested with biotype Y BPH for 48 h.

The combined miRNA-seq and RNA-seq analysis indicated that callose deposition might play a vital role in the rice response to BPH infestation. Callose deposition prevents plant hoppers from ingesting phloem sap (Hao et al., 2008; Du et al., 2009). Specifically, callose deposition ensures that the phloem sieve tubes remain occluded. To determine the role of callose in the rice response to BPH infestation, the expression of genes related to callose synthesis and degradation was examined. Consistent with the RNA-seq results, the transcription of *callose synthase 3*-*like* (*CAL1*) increased, while several *1,3-beta-glucanase* (*BG*) genes decreased, in RY48 (**Figures 7A, B**). To confirm this result, callose deposition was measured during BPH infestation. The outermost sheaths of biotype Y BPH-infested seedlings exhibited larger and more numerous callose spots than those from biotype 1 BPH-infested and uninfested seedlings (**Figures 7C, D**). These results suggest that BPH infestation, particularly infestation with biotype Y BPH, results in the activation of callose synthesis and the suppression of callose degradation.

## 4 Discussion

The management of rice plant resistance to BPH infestation will be crucial for effective pest management. Understanding the defense strategies employed by resistant rice varieties against both avirulent and virulent BPH populations can provide valuable insights for developing effective pest control strategies. Transcriptomics, which can quantify changes in gene expression and associated regulatory mechanisms, can aid in unraveling these complexities. In plants, miRNAs are involved in various developmental processes and play significant roles in abiotic and biotic stress responses (Zhang et al., 2013; Kumar, 2014; Zhang et al., 2016; Li et al., 2017; Mangrauthia et al., 2017; Natarajan et al., 2018). Specifically, miRNAs regulate targeted gene expression by binding complementary sequences in mRNA molecules, resulting in degradation and/or inhibited translation (Bartel, 2009; Marin et al., 2010; Peng et al., 2014). Integrated mRNA and miRNA transcriptomics analyses have been used to identify miRNA-mRNA networks associated with developmental processes and insect defense (Tan et al., 2020; Li et al., 2021; Mei et al., 2021). However, little attention has been paid to defense strategies employed by resistant rice against differentially virulent BPH populations. In this study, we employed an integrated mRNA and miRNA transcriptomics approach to characterize the defense responses of resistant YHY15 rice (contains *Bph15*) to infestation with avirulent (biotype 1) and virulent (biotype Y) BPH. Our results revealed that YHY15 rice seedlings exhibited distinct responses under the two infestation scenarios (**Figure 1**). YHY15 rice is highly resistant to biotype 1 BPH and susceptible to biotype Y BPH, which is in accordance with previous reports (Yang et al., 2004; Jing et al., 2011; Guan et al., 2022).

The BPH-resistance gene *Bph15* has been widely applied in rice breeding programs, although the molecular mechanisms underlying *Bph15*-mediated resistance remain unclear (Lv et al., 2014; Zheng et al., 2021). Previous RNA-seq studies of *Bph15* introgression lines and recipient lines before and after BPH infestation have identified key defense mechanisms associated with phytohormone signaling, mitogen-activated protein kinase (MAPK) cascades, receptor kinases, protein post-translational modifications, TFs, Ca^2+^ signaling, and pathogenesis-related proteins (Lv et al., 2014). In addition, 20 upregulated and 3 downregulated miRNAs were identified in resistant rice variety P15 (containing *Bph15*) compared to susceptible rice variety PC (recipient line) (Wu et al., 2017). Combined with the mRNA transcriptome data, the 67 potential targets of these miRNAs were related to resistance responses to avirulent BPH, including abiotic and biotic stimuli, regulation of plant hormones (GA, SA, ET, and CK), cellulose biosynthesis, amino acid biosynthesis, and protein folding (Cheng et al., 2013; Lv et al., 2014; Wu et al., 2017). Here, comprehensive analysis of miRNA-seq and mRNA-seq data shed light on the underlying mechanisms responsible for the contrasting responses of YHY15 rice to BPH biotypes with different virulence.

Plant defense against BPH is a dynamic and sophisticated process which involves many levels of organizational and functional complexity (Barah and Bones, 2015; Erb and Reymond, 2019). During feeding, the BPH stylet transiently punctures the epidermis and then penetrates the plant cell wall. Subsequently, the insect salivates into the cells and ingests the phloem sap (Hao et al., 2008). According to electronic penetration graph (EPG) waveform recordings, BPH begin feeding on phloem sap within 1-3 hours of settling on rice plants (Hao et al., 2008). A greater number of miRNAs were found to be upregulated in biotype 1 BPH-infested resistant P15 rice (a *Bph15* introgression line) than in susceptible PC rice (recipient line) during the early infestation stage (6 h) when the plants had not yet been severely damaged (Wu et al., 2017). In another report, the inducible BPH defense responses (indicated by upregulated DEGs and DEMs) were more robust during the early feeding stages (e.g., 6, 12, and 24 h) in resistant BPH6G rice (*BPH6*-transgenic rice) than in susceptible Nipponbare rice (wild type, WT) (Tan et al., 2020). Moreover, a miRNA profiling was conducted on resistant IR56 rice (carrying *Bph3*) under separate infestations of a virulent IR56-BPH and an avirulent TN1-BPH (Nanda et al., 2020). This study revealed that BPH feeding caused significant alterations in miRNA expression profiles of IR56 rice, with a greater number of miRNAs showing downregulation when IR56 rice was infested with TN1-BPH. However, the distinct mechanisms underlying rice plant responses to BPH of varying levels of virulence remains unclear. Here, resistant YHY15 rice plants were exposed to avirulent (biotype 1) and virulent (biotype Y) BPH. Notably, DEMs were only identified in rice plants infested with biotype Y BPH (**Figure 2A**). In addition, many more DEGs were identified in rice plants infested with biotype Y BPH, regardless of the infestation time (**Figure 2E**). Together, these results suggest that biotype Y BPH elicit more intense defense responses in YHY15 rice. DaEGs related with cytochrome P450 (KEGG) were more common at the early infestation stage (6 h) in rice infested with both types of BPH (**Figure 2F**; **Supplementary Figures 1A, B**). At the later infestation stage (48 h), overlapping DEGs in R0 *vs* RT48 and R0 *vs* RY48 were enriched in photosynthesis-related pathways (**Figure 2F**; **Supplementary Figures 3A, B**). Specifically, DEGs in R0 *vs* RY6 were mainly enriched in photosynthetic organisms and DNA replication proteins (**Figure 2F**; **Supplementary Figures 2A, B**), indicating that damage to the photosynthetic system occurs earlier in biotype Y BPH-infested rice. Taken together, compared with biotype 1 BPH, infestation with biotype Y BPH results in more serious damage and induces more intense transcriptional responses in YHY15 rice.

In plants, including rice, phytohormone signaling is widely known to be involved in insect defense and resistance (Liu et al., 2016; Jing et al., 2023; Liu Q. et al., 2021). Among phytohormones, JA is critical to the regulation of plant defenses against insect herbivores (Thaler et al., 2012; Howe et al., 2018). Other phytohormones, such as SA, ET, BR, ABA, and CK are also involved in plant responses to herbivory through cross-talk with JA signaling (Bruessow et al., 2010; Schäfer et al., 2015; Pan et al., 2018; Ma et al., 2020; Li et al., 2022; Zhang et al., 2022). In this study, we found that infestation with differentially virulent BPH populations resulted in noticeable effects on the expression of genes associated with phytohormone signaling in YHY15 rice plants. Specifically, infestation with biotype 1 BPH induced JA- and ABA-related signaling pathways, but suppressed ET- and BR-related signaling (**Figure 5**). While, infestation with biotype Y BPH induced more moderate expression of JA- and ABA-related pathways. These results suggest that although infestation with virulent biotype Y BPH results in extensive damage to YHY15 rice plants, they exhibit weaker JA- and ABA-mediated defense responses. These findings are in line with previous research suggesting that IR56 BPH can overcome *Bph3*-mediated resistance by suppressing the transcription of defense-responsive MAPK pathways, phytohormone biosynthesis, and secondary metabolite production (Nanda et al., 2018). Previous studies also suggest that oral secretions, digestive and detoxifying enzymes, and endosymbionts can help insect herbivores adapt to host plants (Simon et al., 2015; Yates and Michel, 2018). We therefore speculate that specific BPH effectors may interact with *Bph15* and affect *Bph15*-mediated immunity in rice. However, further studies will be required to test this hypothesis.

The DEMs and their associated mRNAs identified in this study may potentially play roles in the response of rice to BPH infestation. Among the identified DEMs, miR156, miR5076, miR1856, miR398, miR5072, miR5079, miR408, miR2873, and miR169 exhibited significant variation in amplitude (**Supplementary Table 3**). In addition, many of them have been implicated in various plant developmental processes and stress responses (Li et al., 2017b) (Sharma et al., 2015; Zhao et al., 2017; Lin et al., 2018; Liu et al., 2019; Liebsch and Palatnik, 2020; Li et al., 2020; Liu X. et al., 2021; Gao et al., 2022; Pachamuthu and Hari Sundar, 2022; Zhao et al., 2022). The altered expression of these miRNAs may enhance BPH resistance in YHY15 rice. Combined with mRNA transcriptome data (**Table 2**), we found that the miR156-JA, miR396b–GRF8–F3H–flavonoid, and miR398b-callose deposition pathways may contribute to the resistance of YHY15 rice to biotype Y BPH. Physiological tests verified the increased deposition of callose in biotype Y BPH-infested rice (**Figure 7**). Notably, these miRNA-mediated responses only occurred in rice infested with biotype Y BPH, which suggests that they may contribute to the differential resistance of rice against BPH populations with varying levels of virulence.

Finally, research suggests that numerous TFs are involved in the rice response to BPH infestation. For example, OsMYB30 (an R2R3 MYB TF) induces the expression of phenylalanine ammonia-lyase (PAL) enzymes, thereby improving BPH resistance in rice (He et al., 2020). OsMYB22 promotes rice resistance by affecting flavonoid biosynthesis (Sun et al., 2023). The bHLH protein MYC2 is involved in JA-mediated insect resistance (Schweizer et al., 2013; Xu et al., 2021). OsWRKY45, OsWRKY53, OsWRKY70, and OsWRKY89 also mediate herbivore resistance (Chujo et al., 2007; Wang et al., 2007; Hu et al., 2015; Li et al., 2015; Huangfu et al., 2016). A recent study has demonstrated the pivotal involvement of OsWRKY71 in *Bph15*-mediated resistance (Li et al., 2023). Here, we identified several differentially expressed TFs associated with BPH infestation. Notably, several predominant TF families, including bHLH, MYB, ERF, WRKY, bZIP, NAC, C2H2, TALE, G2-like, HD-ZIP, MYB-related, HSF, and NF-Y, were differentially responsive to BPH infestation. The expression of most TFs was altered in biotype Y BPH-infested rice, indicating their specific roles in defense against the virulent biotype. Notably, the expression of certain other TFs was disturbed specifically in response to biotype 1 BPH infestation. These findings highlight the important functions of TFs in BPH defense and warrant further research to uncover their specific roles.

## 5 Conclusion

In conclusion, our study provides valuable insights into the differential defense strategies employed by resistant YHY15 rice (carrying BPH resistance gene *Bph15*) against avirulent (biotype 1) and virulent (biotype Y) BPH. The BPH defense response was found to involve the modulation of miRNAs, TFs, phytohormone signaling pathways, and the induction of callose deposition. These responses were most noticeable in biotype Y BPH-infested rice plants. These findings contribute to the elucidation of the molecular intricacies underlying rice-BPH interactions and pave the way for further research into the specific genes, pathways, and regulatory elements involved in plant defense against diverse BPH populations. Studying these defense mechanisms will aid our understanding of the intricate interactions between rice and BPH and allow the development of targeted pest control strategies for improved rice cultivation. It is worth noting that the infestation-induced defense responses of YHY15 rice do not appear to alter the survivability of biotype Y BPH, implying the existence of corresponding adaptive responses in the virulent biotype. This result will be the subject of in-depth exploration in future studies.

## Data availability statement

All raw RNA sequencing data generated in this study have been deposited under the NCBI SRA database under BioProject PRJNA997052 and PRJNA994598.

## Ethics statement

The manuscript presents research on animals that do not require ethical approval for their study.

## Author contributions

BY: Writing – original draft, Writing – review & editing. MG: Investigation, Writing – original draft, Writing – review & editing. YX: Investigation, Writing – review & editing. QY: Writing – review & editing. BL: Formal analysis, Writing – review & editing. ML: Writing – review & editing. YS: Writing – review & editing. CL: Writing – review & editing. JX: Writing – review & editing. JL: Writing – review & editing. WH: Writing – review & editing. HT: Writing – review & editing. PL: Writing – review & editing. QL: Investigation, Supervision, Formal analysis, Writing – review & editing. SJ: Supervision, Project administration, Resources, Funding acquisition, Writing – original draft, Writing – review & editing.
