# Supplementary material for: Combined miRNA and mRNA sequencing reveals the defensive strategies of resistant YHY15 rice against differentially virulent brown planthoppers
Source: Front Plant Sci. 2024 Mar 18;15:1366515. doi: 10.3389/fpls.2024.1366515 (PMC10982320; doi:10.3389/fpls.2024.1366515)

Supplementary Figure 1. KEGG pathway enrichment analyses (**A**) and GO function analyses (**B**) of DEGs overlapped in R0 vs RT6 and R0 vs RY6.

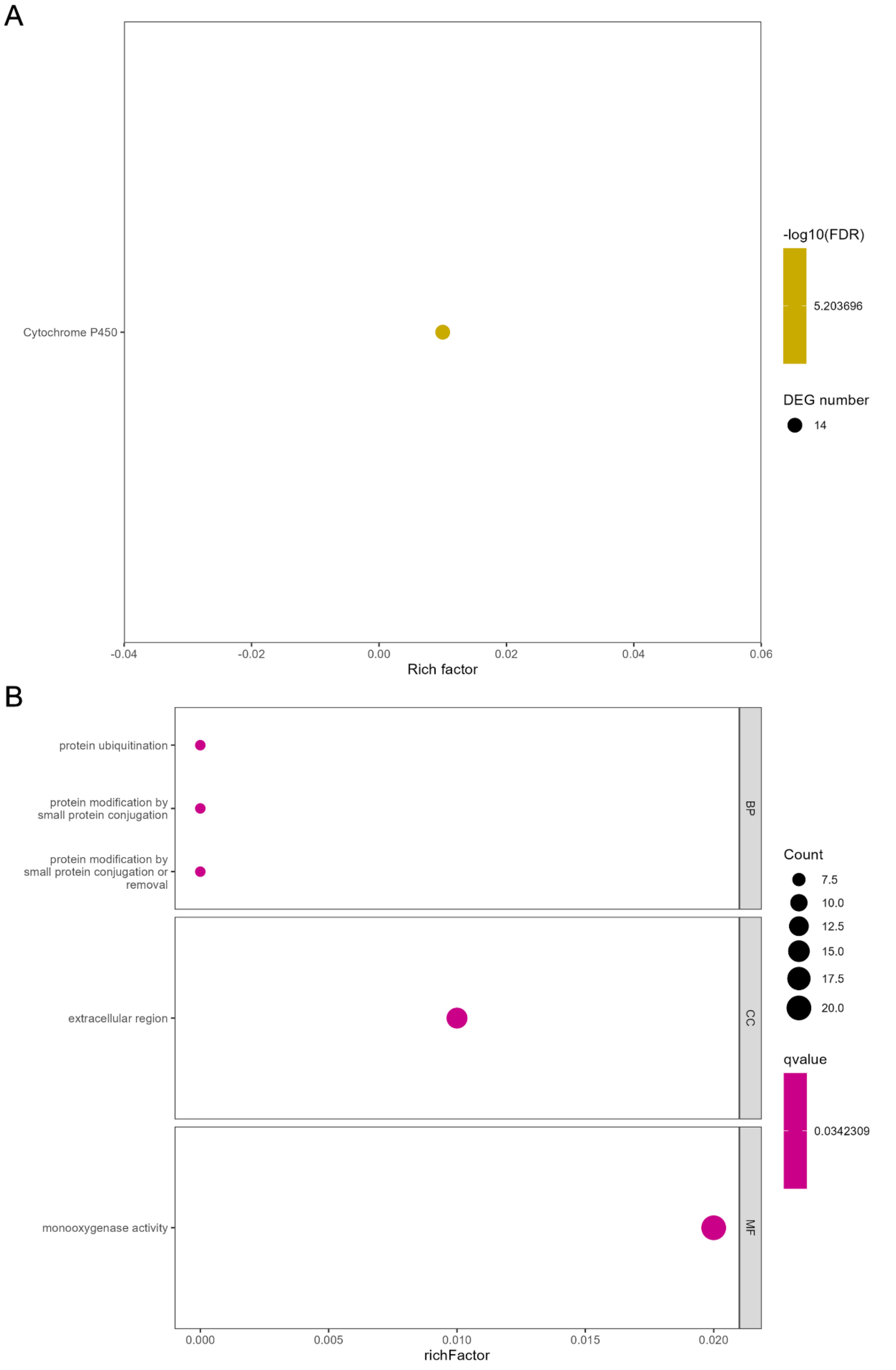

Supplementary Figure 2. KEGG pathway enrichment analyses (**A**) and GO function analyses (**B**) of DEGs specifically in R0 vs RY6.

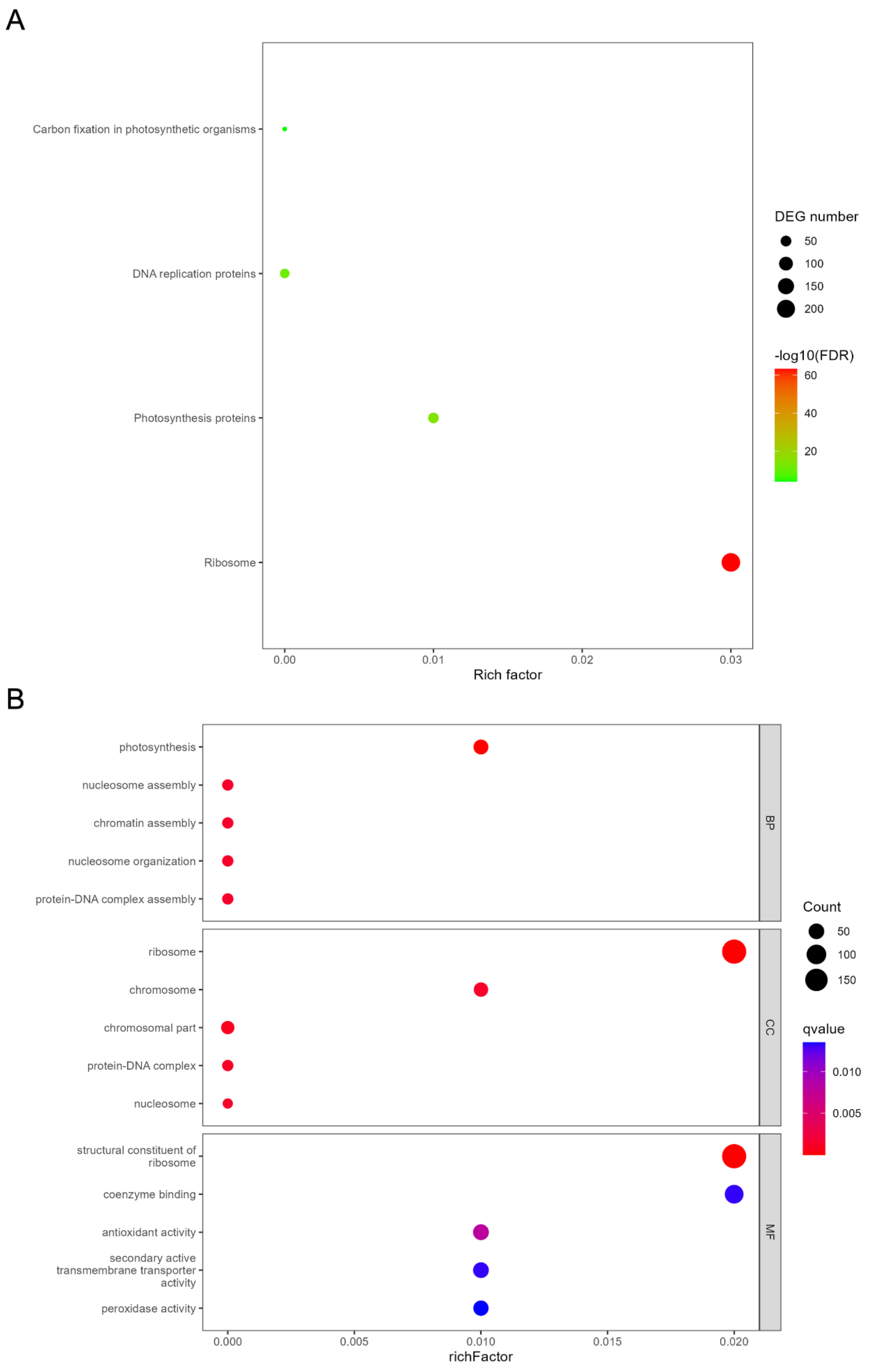

Supplementary Figure 3. KEGG pathway enrichment analyses (**A**) and GO function analyses (**B**) of DEGs overlapped in R0 vs RT48 and R0 vs RY48.

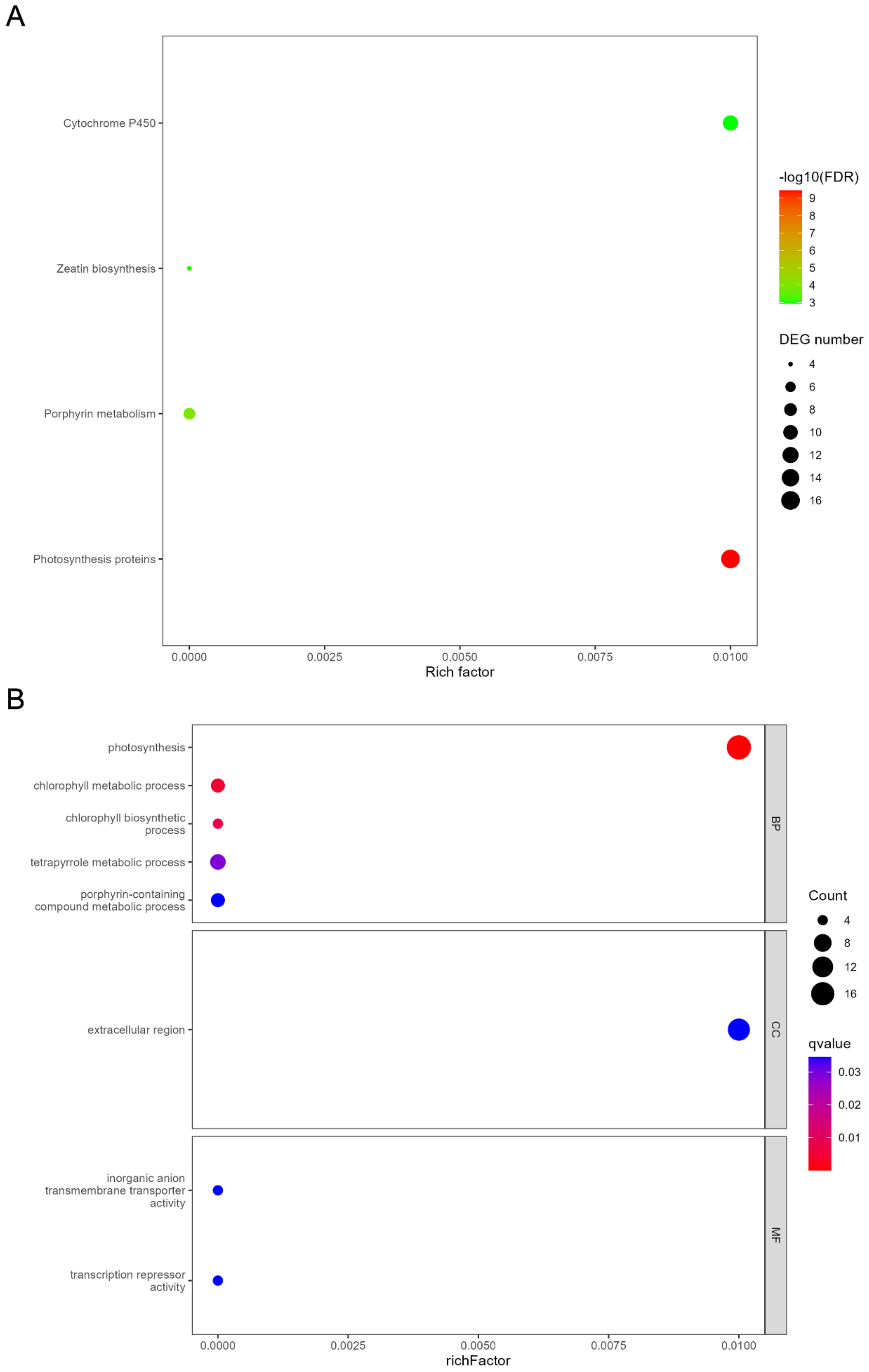

Supplementary Figure 4. KEGG pathway enrichment analyses (**A**) and GO function analyses (**B**) of DEGs specifically in R0 vs RY48.

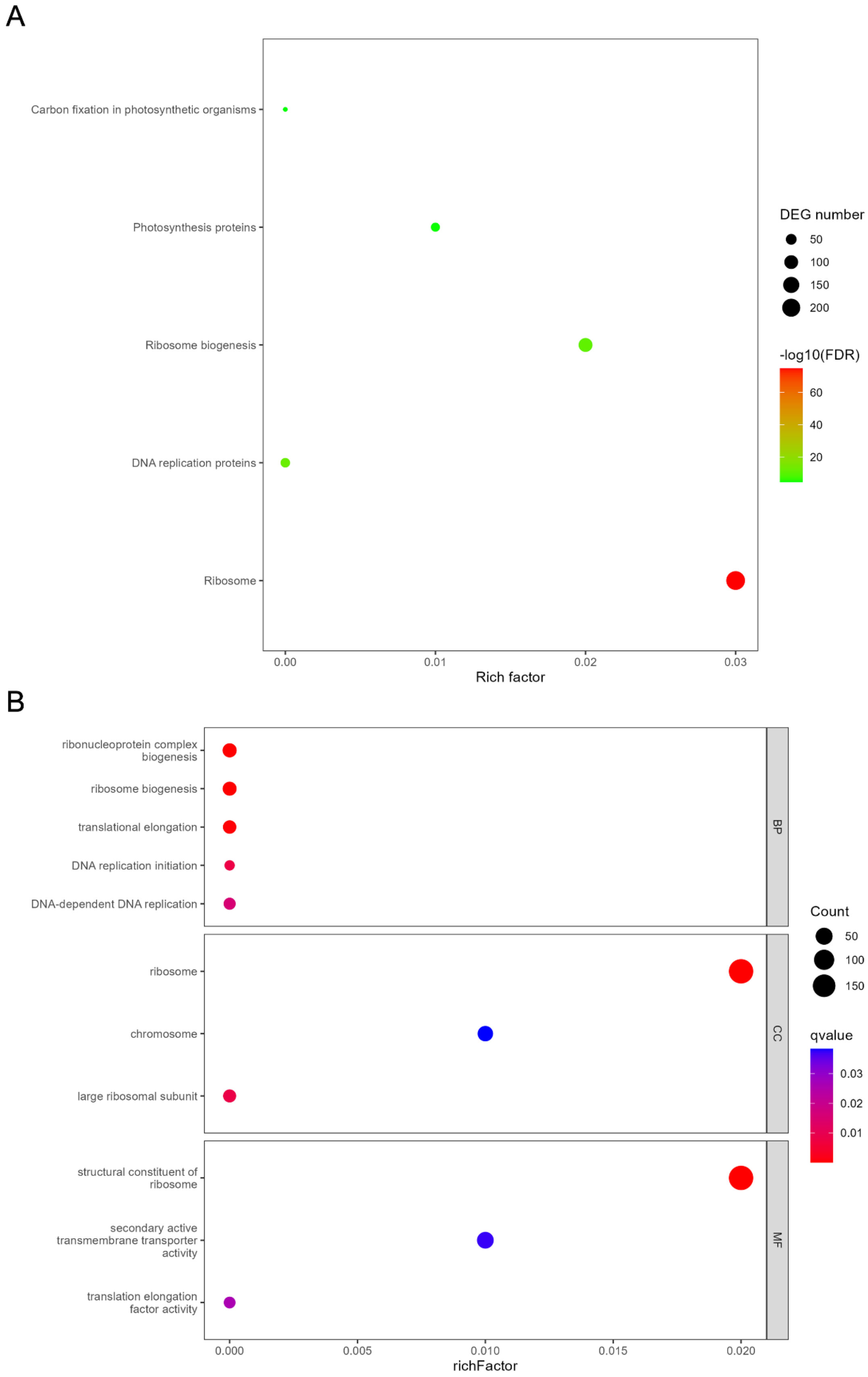

Supplementary Figure 5. GO function analyses of cluster 1 (**A**), 6 (**B**), 7(**C**), 10(**D**), 11 (**E**), and 17 (**F**), respectively.

Supplementary Figure 5A. GO function analyses of cluster 1.

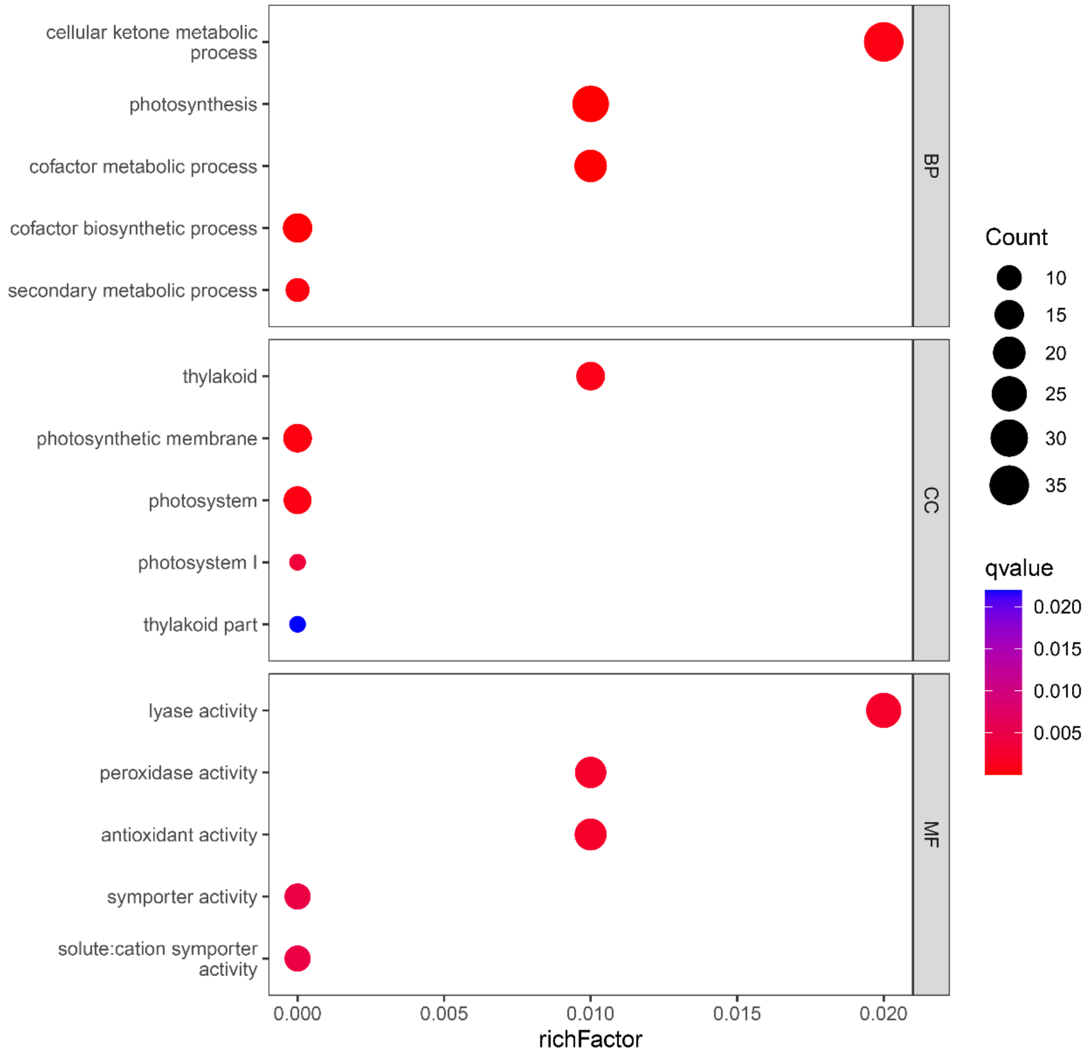

Supplementary Figure 5B. GO function analyses of cluster 6.

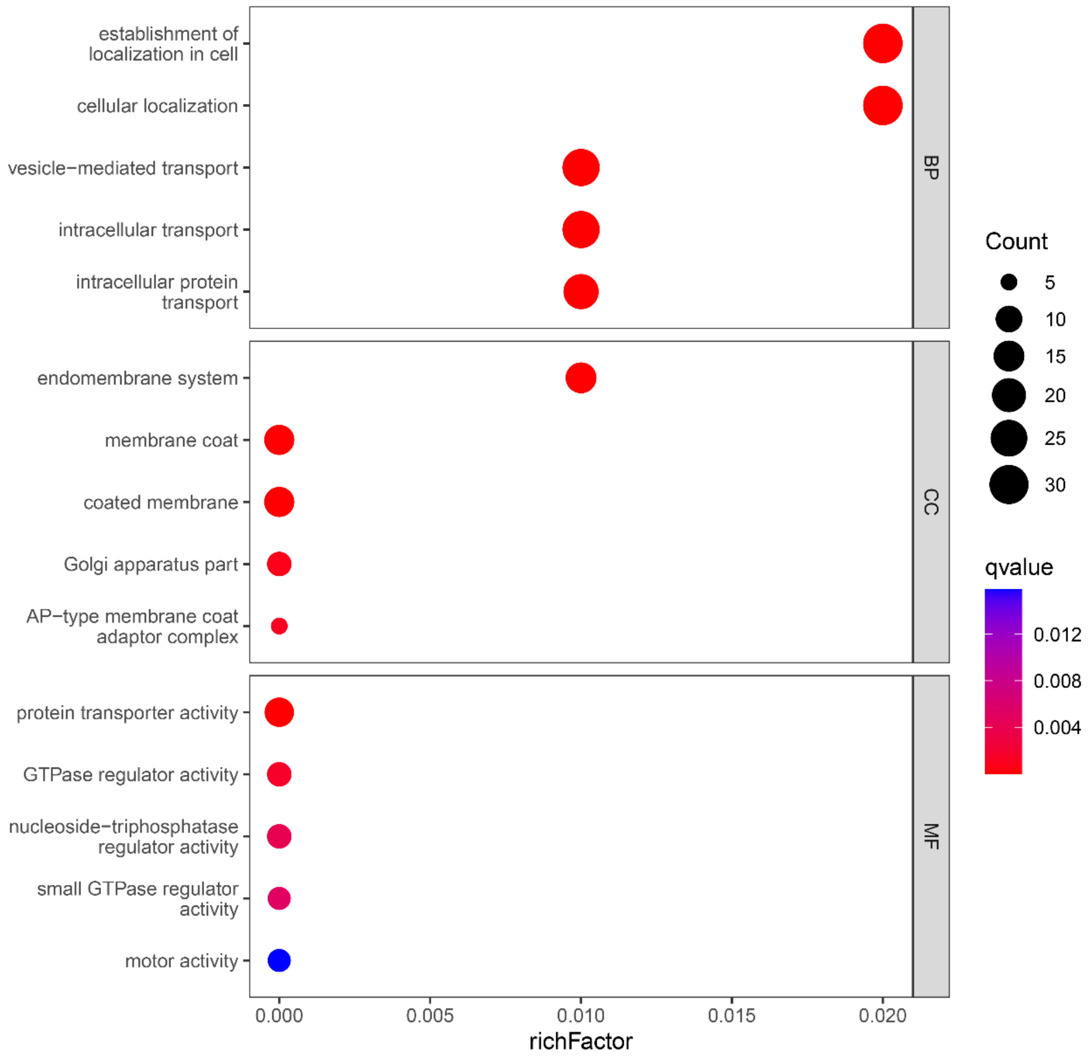

Supplementary Figure 5C. GO function analyses of cluster 7.

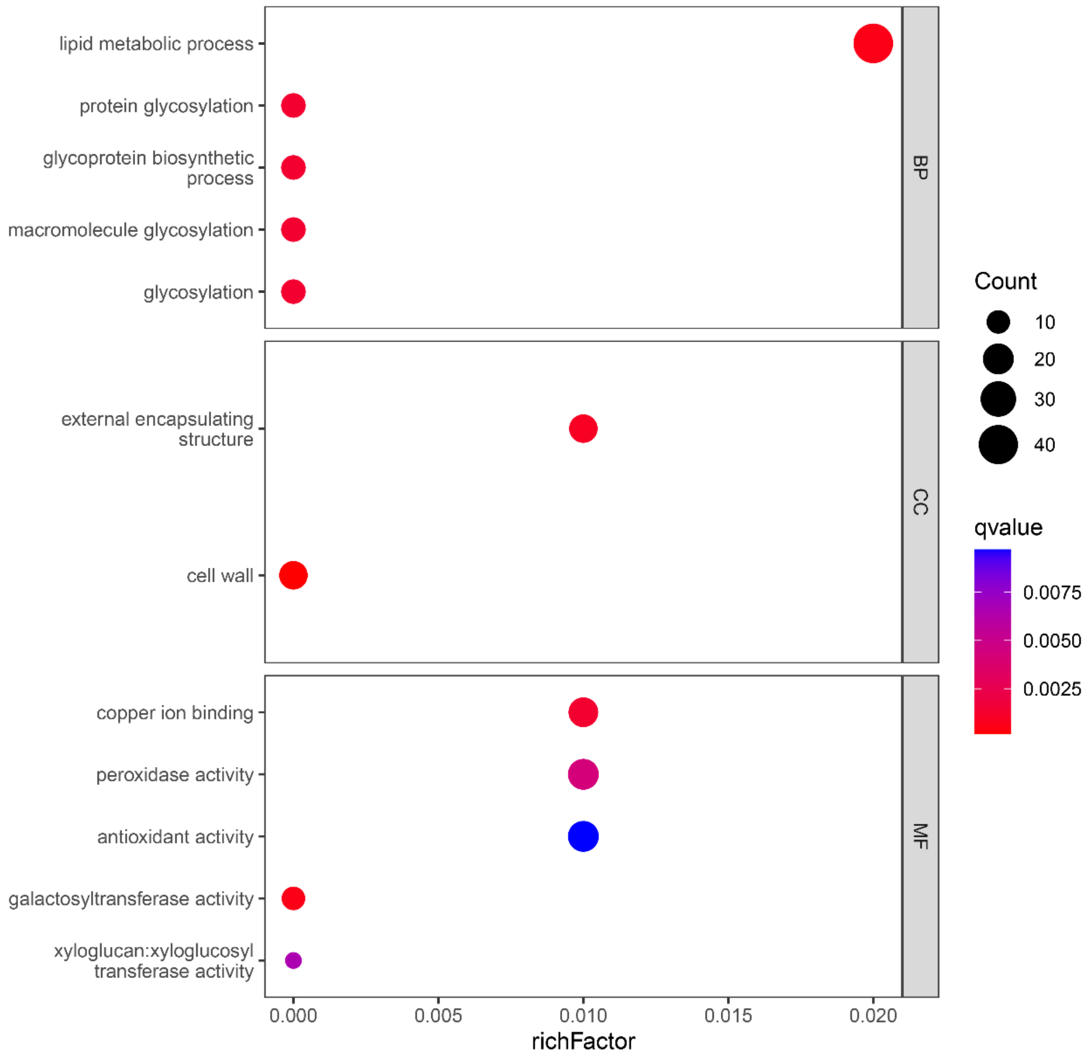

Supplementary Figure 5D. GO function analyses of cluster 10.

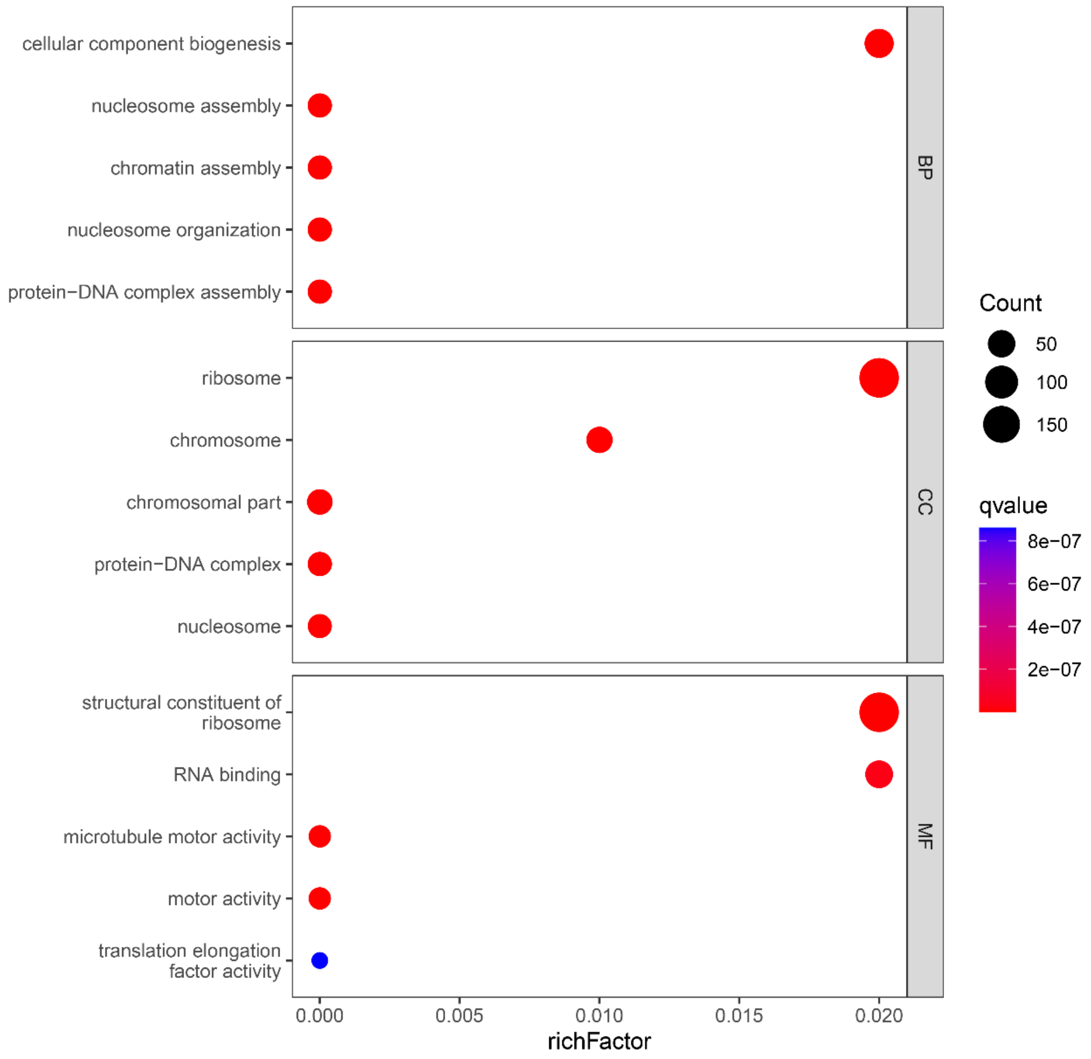

Supplementary Figure 5E. GO function analyses of cluster 11.

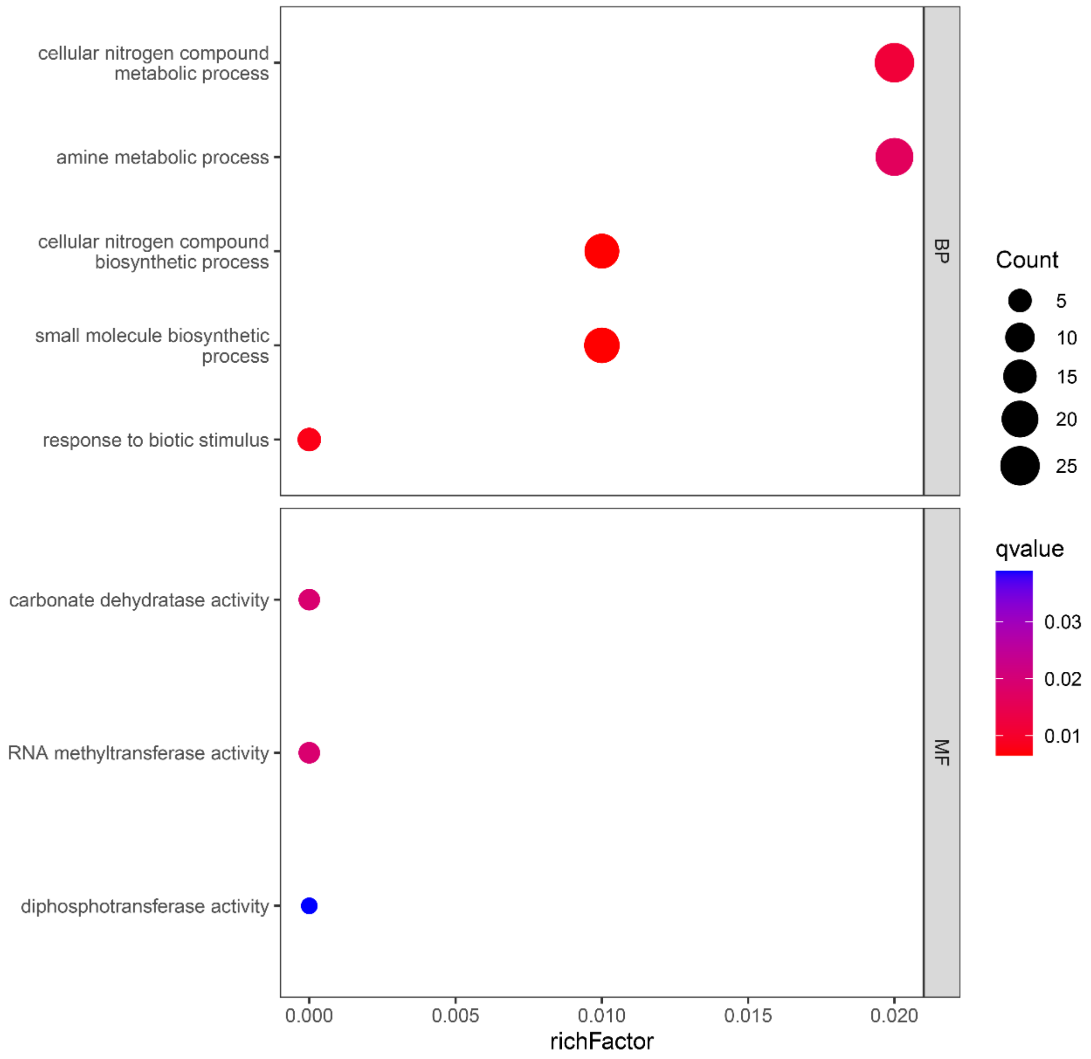

Supplementary Figure 5F. GO function analyses of cluster 17.

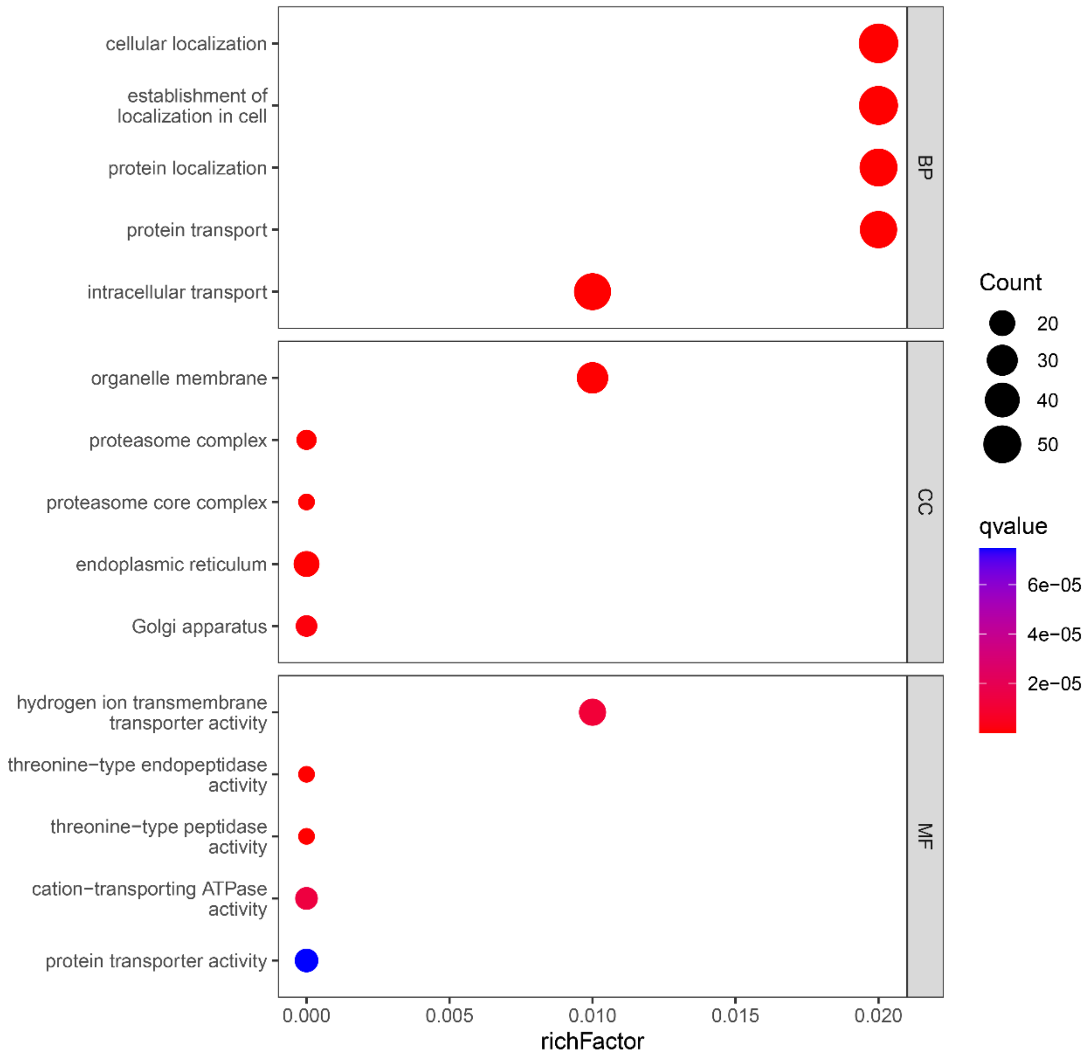

Supplementary Figure 6. Heat map of the expression of genes encodes transcription factors.

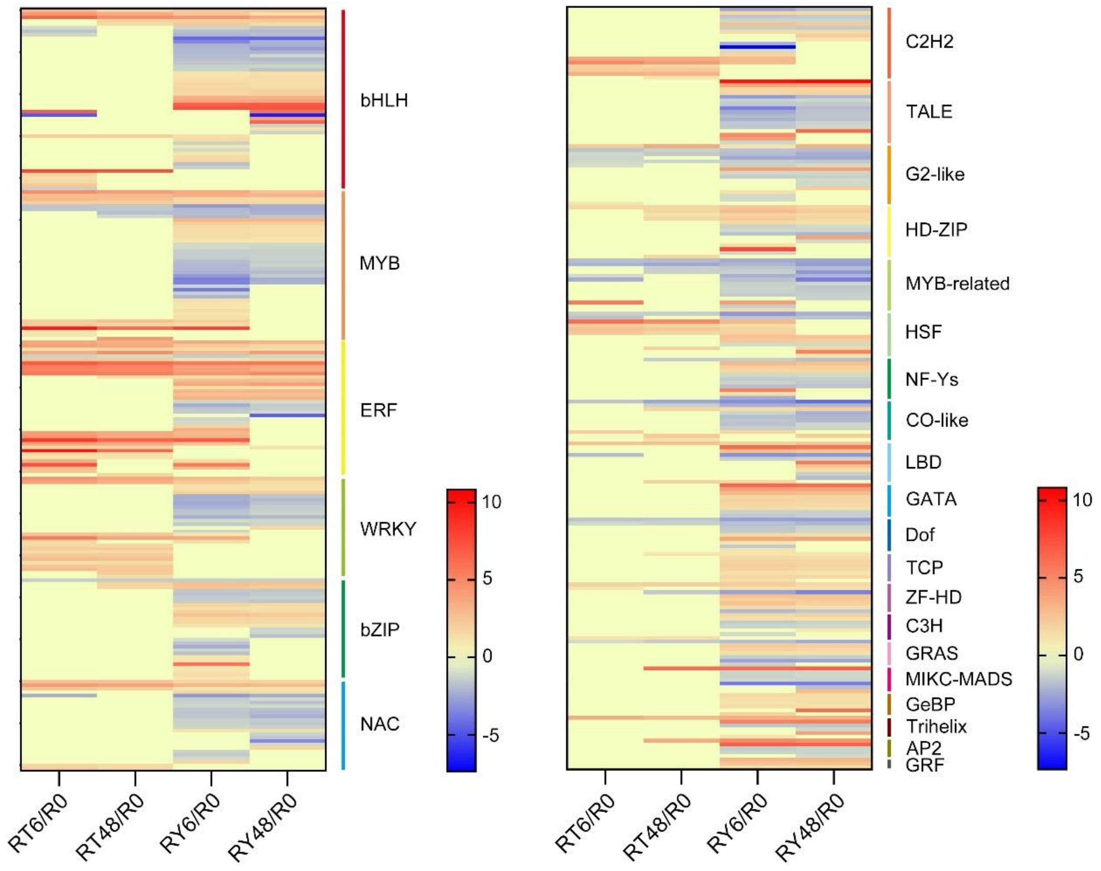

Supplement: Supplementary file 1 [file DataSheet_1.pdf]
